# Characterization of swelling gradients and evaporation dynamics in polymer brushes using high-resolution colorimetry

**DOI:** 10.1140/epje/s10189-026-00613-8

**Published:** 2026-07-30

**Authors:** Vincent Siekman, Sander Reuvekamp, Enqing Liu, Sissi de Beer, Frieder Mugele

**Affiliations:** 1 Department of Chemical Engineering, University of Twente, Drienerlolaan 5, Enschede, 7522 NB The Netherlands; 2 Department of Molecules & Materials, University of Twente, Hallenweg 15, Enschede, 7522 NB The Netherlands

## Abstract

**Abstract:**

We present a white-light interferometry method enabling real-time, full-field measurement of local film thickness with nanometer vertical resolution using a simple optical microscopy set-up. We apply this approach to investigate droplet-induced swelling and vapor-infused deswelling of poly(lauryl methacrylate) (PLMA) brushes in contact with *n*-alkanes (*C*12, *C*14, *C*16), spanning two orders of magnitude in vapor pressure. For uniformly pre-swollen brushes, we demonstrate that evaporation is governed by vapor-phase transport rather than transport within the brush, remaining remarkably constant over swelling ratios from 4.2 to 1.6. Rescaling time by vapor pressure collapses all deswelling data onto a single master curve, consistent with diffusion-limited gas-phase transport. For droplet-induced swelling on initially dry brushes, we quantify the coupled effects of imbibition, evaporation, and condensation on the formation of swelling halos. More volatile solvents produce faster-growing halos that reach a limited lateral extent in open air. The final width follows from a balance between near-constant liquid influx and size-dependent evaporative outflow. Temporal swelling profiles collapse onto a common functional form, indicating universal behavior governed by solvent transport and evaporation. These results establish colorimetric interferometry as an accessible tool for probing dynamic soft interfaces and a means to constrain key model parameters, notably the grafting density σ and the interaction parameter χ, enabling systematic extraction of material properties from dynamic experiments.

**Graphical abstract:**

Schematic illustration of a swelling gradient (“halo” emerging after placing an alkane drop onto an initially dry PLMA polymer brush. The spatiotemporal evolution of the halo region is monitored using white-light colorimetry (inset, top view), enabling quantitative conversion of interference colors into local brush thickness

**Figure:**
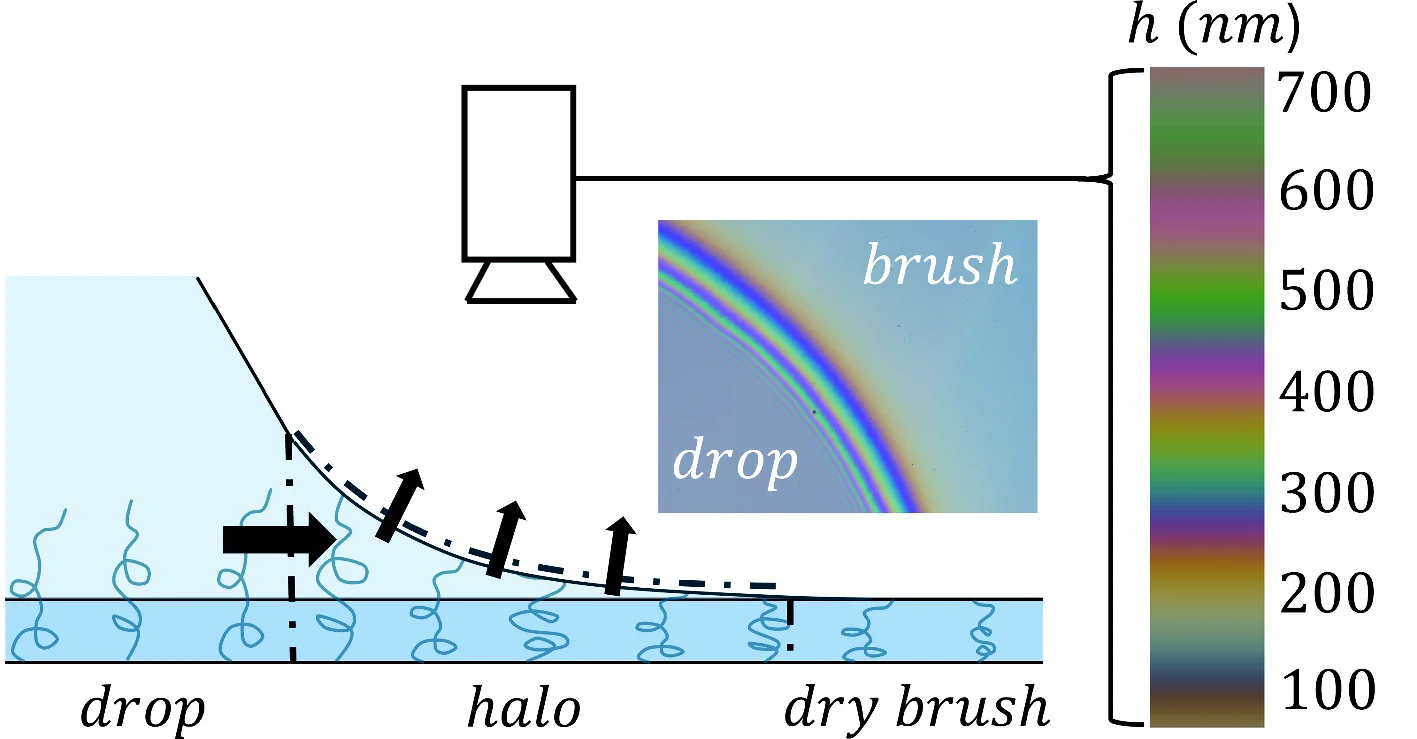

**Supplementary Information:**

The online version contains supplementary material available at 10.1140/epje/s10189-026-00613-8.

## Introduction

Adaptive and responsive surfaces are characterized by their ability to dynamically alter their interfacial properties in response to external stimuli such as solvent composition, altering surface topology [1], temperature [2], or vapor environment [3–5]. Examples include porous materials that regulate liquid transport [6], liquid-infused surfaces [7] with low contact line friction [8], and molecular or polymer coatings [9] that reversibly switch wettability [10]. Polymers are particularly well suited for such applications due to their structural flexibility and strong responsiveness to environmental conditions and are therefore widely used in adaptive surface design [11–14]. In these systems, transport and material response are intrinsically coupled: solvent uptake modifies the structure of the material, while the evolving structure in turn affects further transport. A central challenge is to quantitatively relate local solvent transport to spatially and temporally evolving material responses.

Understanding such coupled dynamics is essential for applications ranging from sensing [13, 15] and actuation [16] to lubrication [17–20] and wetting control [21–24], where the response time and spatial extent of adaptation determine functionality. However, this coupling complicates the classical wetting descriptions, which assume rigid substrates and describe dynamics through surface energy minimization and viscous dissipation near the contact line [25, 26].

When the substrate itself is responsive, additional physical processes arise. Liquid contact introduces new timescales associated with viscoelastic relaxation, solvent transport within the material, and in turn altered wettability [23, 27, 28]. Soft polymer solids like gels, films, and brushes can deform near the contact line [29–32], forming wetting ridges [33, 34]. Polymer brushes, in particular, swell strongly in the presence of solvents driven by differences in chemical potential [11], altering their thickness and mechanical properties [11–14]. This leads to a coupled evolution of the liquid shape and substrate profile, as captured in recent experimental and theoretical descriptions of drops on adaptive substrates [35–38].

To understand and ultimately control such adaptive surfaces, it is necessary to consider both (i) the equilibrium response of the material to solvent uptake and (ii) the kinetics of solvent transport and redistribution, which frequently involve vapor-mediated processes. While qualitative features of such coupled dynamics are often readily identified, achieving a quantitative description remains challenging due to the various interacting processes, which require multiple, and sometimes not well-known, material parameters in existing models [36, 38]. Systematic experimental studies across a broader range of conditions are therefore essential to constrain these models and identify the dominant transport mechanisms [39]. Polymer brushes provide an ideal model system in this context, as they can be synthesized with well-defined thicknesses, controlled chemical composition, and high lateral homogeneity [40, 41]. Their covalent grafting ensures strong anchoring of individual chains, limiting degrafting even under swelling conditions [42, 43], while their strong solvent response enables direct coupling between wetting and substrate dynamics [11, 12, 44].

**Fig. 1:**
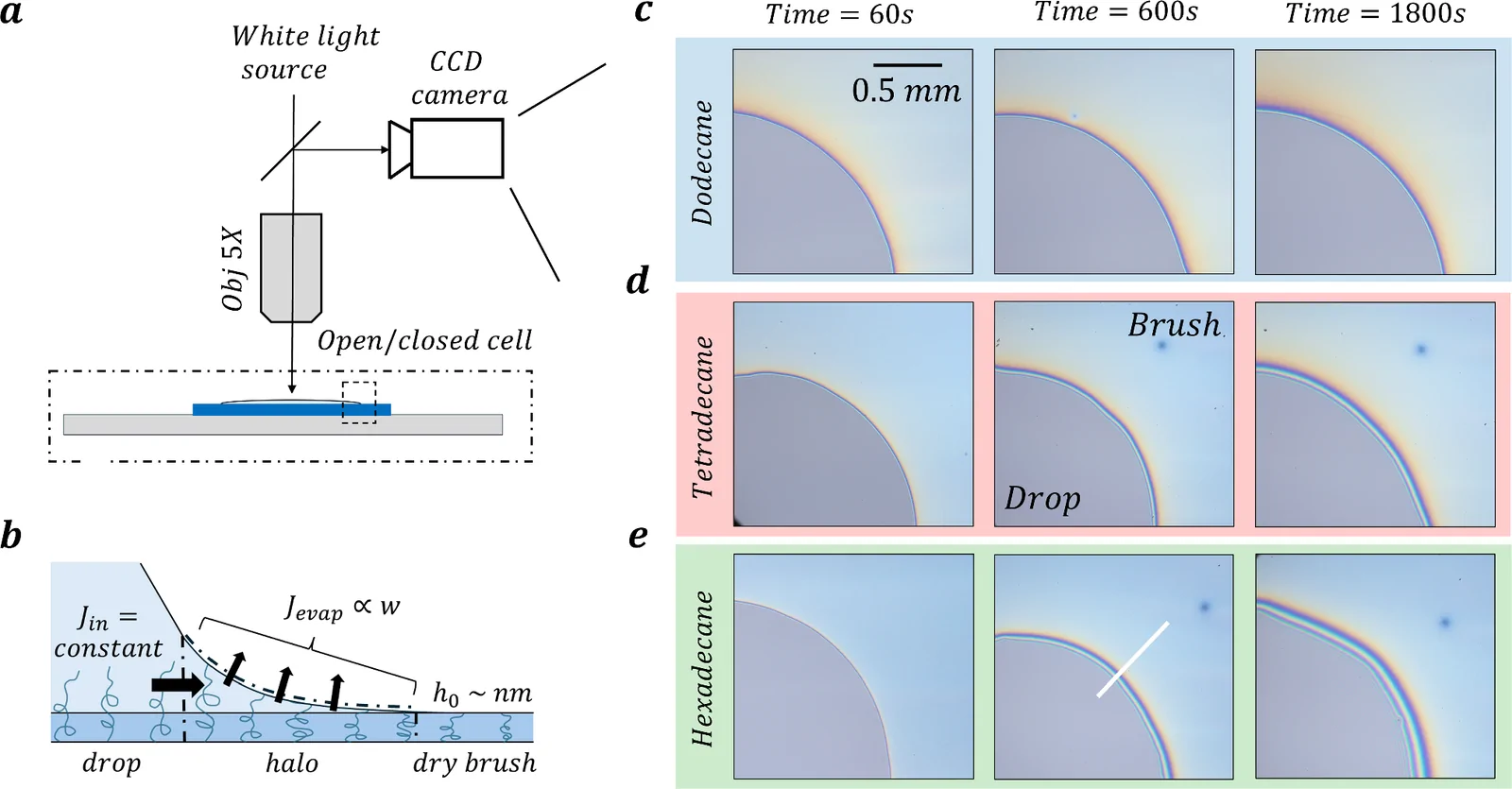
**a** Schematic of the experimental set-up used to study the evolution of brush swelling upon placing an *n*-alkane droplet onto an initially dry PLMA polymer brush layer with thickness of $h_0\approx$160 nm. The process is observed from above using a microscope (5× objective) and recorded via a CCD camera. **b** Cartoon illustrating the lateral spreading of a ‘halo’ normal to the contact line of the drop (white line, panel **e**). Note how the brush swells only several hundred *nm* vertically, while the solvent spreads nearly a *mm* into the brush. The spreading behavior toward the final halo profile depends on the bulk liquid used, as shown for **c** dodecane, **d** tetradecane, and **e** hexadecane. These top-view images show the halo development with the droplet located in the bottom-left corner at $t = 60, 600, \text { and } 1800\text { s}$ in an open-cell configuration. For longer *n*-alkanes, the halo develops more slowly but spreads further

In previous works, we demonstrated that this coupling can give rise to rich non-equilibrium phenomena, including the formation of long-range swelling gradients induced by droplets [39] and thermally activated swelling and wetting transitions [2]. However, capturing the full quantitative kinetic picture of solvent transport remains experimentally challenging, as it is encoded in the spatiotemporal evolution of the swelling profile: spatial gradients reflect solvent fluxes within the brush, while temporal evolution reveals the relative roles of imbibition, evaporation, and condensation.

A major limitation is the lack of techniques combining high spatial and temporal resolution. Methods such as confocal microscopy, ellipsometry, monochromatic interferometry, and atomic force microscopy (AFM) either lack temporal resolution, require sequential scanning, or are limited in spatial coverage. As a result, a complete time-resolved picture of in situ thickness profiles across relevant length scales remains difficult to obtain. To address this limitation, we introduce a colorimetric approach that enables full-field imaging of surface profiles with high temporal resolution.

The paper is organized into three parts. First (section 2), we describe the synthesis and characterization of oleophilic poly(lauryl methacrylate) (PLMA) polymer brushes, which serve as a model system for adaptive surfaces with applications in lubrication and responsive coatings [2, 18, 20].

Second (section 3), we present a detailed quantitative colorimetric method that enables real-time extraction of local film thickness using white-light optical microscopy. By exploiting thickness-dependent interference, this approach achieves nanometer-scale vertical resolution while retaining a large field of view at the high acquisition rate of the camera (Fig. 1a).

Third (section 4), we apply this approach to investigate the swelling response of PLMA brushes to a series of *n*-alkanes spanning a hundredfold difference in saturation pressures, namely dodecane (C$_\textrm{12}$H$_\textrm{26}$), tetradecane (C$_\textrm{14}$H$_\textrm{30}$), and hexadecane (C$_\textrm{16}$H$_\textrm{34}$). We first examine the evaporation of uniformly pre-swollen brush layers. Quantitative agreement with a simple diffusion model provides a constraint on feasible combinations of the interaction parameter (χ) and grafting density (σ), two key parameters for modeling any polymer brush system [38]. We then evaluate droplet-induced swelling on initially dry brushes (Fig. 1c,d,e). Driven by the dynamic coupling between solvent imbibition ($J_{\text {in}}$) and integrated evaporation loss ($J_{\text {evap}}$) (Fig. 1b), the swelling profiles collapse onto a common form with a decay length reminiscent of classical Washburn spreading. Together, these results offer a deeper understanding of the transport properties regulating polymer brush swelling.

## Synthesis and characterization of PLMA brushes

### Synthesis of PLMA brushes

#### List of materials

Native oxide silicon wafers (100 ± 0.5 mm diameter, 525 ± 25 μm thickness, boron-doped, <100> orientation, 5-10Ωcm, OKMETIC) were cut into 1x1 cm pieces and used as a substrate for polymer brush growth. The polymerization was performed in 5 mL (40x20mm, fisherbrand) snap cap glass vials. (3-Aminopropyl) triethoxysilane (APTES, 99%, Sigma-Aldrich), triethylamine (TEA, >99.5%, Sigma-Aldrich), α-bromoisobutyryl bromide (BiBB, >98.0% TCI chemicals), L-ascorbic acid (AA, >99%, Sigma-Aldrich), *N,N,N’,N”,N”*-pentamethyldi-ethylenetriamine (PMDETA, 99%, Sigma-Aldrich), CuCl$_2$ (97%, Sigma-Aldrich), dodecyl methacrylate (12MA, 96%, Sigma-Aldrich), ethanol (absolute ≥99%, Fisher), acetone (>99.5% technical, Boom), toluene (HPLC grade, VWR chemicals), *n*-hexadecane (ReagentPlus 99%, Sigma-Aldrich), *n*-tetradecane (99+%, Alfa Aesar), *n*-dodecane (analytical standard, Sigma-Aldrich) were purchased and used without further purification.

#### Surface anchoring and polymerization

**Fig. 2:**
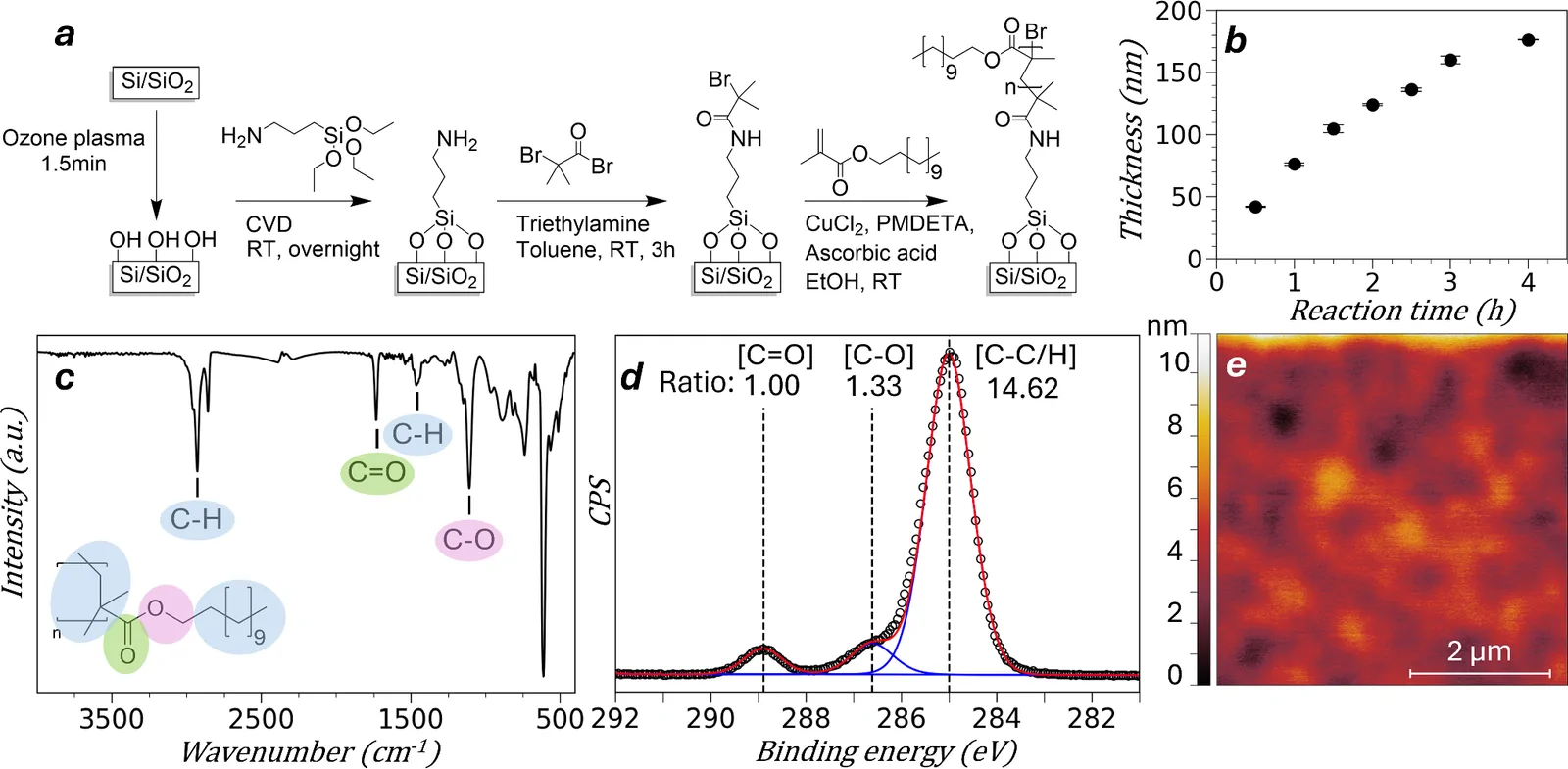
a) Synthesis scheme of poly-lauryl methacrylate brushes. The brushes were characterized by ellipsometry to yield b) the kinetics of brush growth, mostly linear up until 200 nm. Error bars represent the standard error of the mean of two samples, c) FTIR transmission spectrum, d) XPS, of which the narrow C1s scan is shown with the XPS signal (open circles), fits (blue), and fits superimposed (red), e) AFM tapping mode topography scan on a 173 nm thick brush

The poly-lauryl methacrylate (PLMA) polymer brushes were synthesized via Surface-Initiated Activators ReGenerated by Electron Transfer for Atom Transfer Radical Polymerization (SI-ARGET-ATRP)  [45, 46], following protocols adapted from  [39] and [47], see Fig. 2a for a schematic overview. This approach enables the formation of densely grafted polymer brushes with well-controlled chain length [40], while operating at low catalyst concentrations and maintaining tolerance to oxygen [48].

As an anchoring agent, we use (3-aminopropyl) triethoxysilane (APTES), which is known for its convenient coupling with hydroxyl groups and higher stability than other trifunctional silanes [42]. Although APTES is known to hydrolyze upon exposure to water [49], in our hydrophobic system this does not pose an issue since water has limited penetration into the brush, ensuring long-term stability [43]. First, the silicon wafers were cleaned by sequential sonication for 1 min in acetone, ethanol, and finally repeatedly with demiwater, after dried under a nitrogen stream. Subsequently, the substrates were subjected to ozone plasma treatment for 1.5 min to activate the interfacial hydroxyl groups. The activated wafer pieces were immediately placed into a desiccator around a Petri dish containing APTES (0.1 mL, 0.43 mmol). The desiccator was evacuated for 10 min and subsequently closed, allowing overnight vapor deposition.

The amine-terminated substrates were transferred to an ethanol bath for cleaning, rinsed twice with demi-water and ethanol, and blown dry under a nitrogen stream. Together with a stirring bar, the samples were placed face-up into a flat-bottomed reaction vessel. Meanwhile, ice-cooled anhydrous toluene (100 mL) and triethylamine (1.12 mL, 8.05 mmol, TEA) were combined in an Erlenmeyer flask. To minimize gel formation, α-bromoisobutyryl bromide (1.0 mL, 8.09 mmol, BiBB) was added dropwise while stirring vigorously. This solution was transferred to the aforementioned reaction vessel containing the substrates, covered, and allowed to react for 3 h while stirring sufficiently strong that the solution flows over the wafers. The substrates were cleaned by transferring them into a toluene bath, rinsing with toluene, twice with ethanol and demi-water, and drying under a nitrogen stream.

Finally, the PLMA brushes were synthesized in seperate 5-mL snap cap glass vials at room temperature, filled to the top with 7 mL of solution (to minimize the air present above the solution). The following procedure yields 4 vials. Prior to every synthesis, two stock solutions were prepared: (1) L-ascorbic acid (AA, 84 mg, 477 μmol) was dissolved in ethanol (20 mL) by sonication, and (2) CuCl$_2$ (28 mg, 208 μmol) in ethanol (10 mL) by sonication before adding *N,N,N’,N”,N”*-pentamethyldi-ethylenetriamine (PMDETA, 0.1 mL, 0.48 mmol). Into each vial, 4.8 mL of the AA solution (1) was injected, quickly followed by 0.44 mL of the catalyst solution and (2) by dodecyl methacrylate monomer (12MA, 1.75 mL, 6.0 mmol), after which a functionalized wafer was placed face-up into the solution and the vial was quickly sealed to limit oxygen poisoning of the catalyst. The molar ratio of AA/CuCl$_2$/PMDETA/12MA in the reaction mixture was 12.5:1:2.3:655. After the desired polymerization reaction time, the polymer brushes were rinsed twice with toluene, ethanol, and demi-water and blown dry under a nitrogen stream.

### Characterization of PLMA brushes

#### Spectroscopic ellipsometry measurements

Individual dry brush thickness measurements were taken on an M2000-V spectroscopic ellipsometer (J.A. Woolam controlled by CompleteEASE software), operating in the wavelength range of 500–1000nm, with 5-s sampling time at three angles of incidence (65, 70, and 75$^{\circ }$). The data were fitted using the EP4 analysis software-provided dispersion relations in an optical model composed of a semi-infinitely thick silicone substrate, a 3.5-nm-thick SiO$_2$ layer, a Cauchy layer, fitting the first- and second-order parameters and air. Brush thicknesses were recorded for samples of various reaction times, presented in Fig. 2b. The steady increase of brush thickness over time reflects the controlled nature of the ARGET-ATRP polymerizations.

The hexadecane swelling gradient in PLMA, for comparison with the colorimetric method described in 3.1, was assessed using the 2D mapping function on a Spectroscopic Ellipsometer (SE) with Nanofilm-EP3 SE (ACCURION GmbH, Göttingen, Germany) at an angle of incidence of 70.018$^{\circ }$. Psi mapping was performed at various wavelengths (609.1, 709.9, 810.0, 905.1 nm), and subsequently converted to a height profile by custom python software. Here, the measured Ψ values were interpreted using a three-layer optical model consisting of air (n=1.0), a homogeneous polymer layer (with fixed n=1.474, k=0), and a semi-infinite silicon substrate (n=3.88, k=0.02). For each pixel, the thickness was determined by minimizing the least-squares deviation between measured and modeled Ψ values using the Fresnel equations across all available wavelengths. A precomputed lookup table of Ψ as a function of thickness (130–800 nm) and wavelength enabled efficient pixel-wise inversion. The resulting thickness maps were subsequently converted to spatially resolved height profiles using hardware-provided pixel-to-distance scaling factors. An example of such height profile normal to the contact line is plotted in Fig. 4c (black symbols).

#### Fourier transform infrared spectroscopy (FTIR)

To verify successful brush growth by the appearance of characteristic absorbance bands, Fourier transform infrared spectroscopy measurements were taken on a Bruker Alpha spectrometer (see Fig. 2c). The background spectrum was recorded in open air. Absorption bands at 1103 cm$^{-1}$, 1456 cm$^{-1}$, 1728 cm$^{-1}$, and 2923 cm$^{-1}$ are attributed to C–O stretching, C–H bending, C=O stretching, and C–H stretching vibrations, respectively, consistent with the presence of the polymer. The expected atmospheric CO$_2$ band around 2338 cm$^{-1}$ is largely removed by background subtraction.

#### X-ray photoelectron spectroscopy (XPS)

XPS measurements were taken using a VersaProbe II X-ray photoelectron spectrometer. Samples were analyzed using a focused monochromated Al Kα X-ray source (spot size of 100 μm, power 25 W (15 kV)) and passing energy of 23.5 eV for the narrow scans and 187.85 eV for the wide scans. Charge neutralization was applied using the instrument’s dual-beam charge compensation system, employing low-energy electrons (1.5 eV, 20 μA). All spectra were acquired under ultra-high vacuum conditions (base pressure 1.0·10$^{-6}$ Pa) and were processed using CasaXPS. For the narrow spectra, a Shirley background was subtracted and peaks were fitted using symmetric Gaussian–Lorentzian line shapes (GL(30)). All spectra were referenced to the C1s peak attributed to C–C and C–H atoms at 285.0 eV. In Fig. 2d, we observe a ratio of [C-C/H] (285.0 eV): [C-O] (286.6eV): [C=O] (288.9 eV) of 14.62:1.33:1.00. This ratio matches the expected ratio of 14:1:1 of PLMA, confirming the presence of our polymer at the interface. A wide-range spectrum is presented in the Support Information (SI) 1, Fig. S3, further confirming the presence of the polymer and the absence of other contaminants.

#### Atomic force microscopy (AFM)

The dry brush topography was assessed using AFM in tapping mode under ambient conditions, using a Bruker Icon (Santa Barbara, CA, USA) with silicon probes (tip radius r < 8 nm, spring constant k ≈ 0.6 Nm$^{-1}$, HQ: NSC36/Cr-Au BS, MikroMasch). Gwyddion software was used to process and analyze the AFM topography images [50], employing standard processing steps regarding data leveling and background subtraction, including (i) level data by mean plane subtraction, (ii) removing the polynomial background using a vertical polynomial degree of two, and (iii) shifting the minimum data value to zero. A representative topographical image is shown in Fig. 2e for a 173-nm-thick brush sample (5 × 5 μm$^2$ scan area). No distinct surface morphology is observed. The height variation remains limited to approximately 10 nm across the scan, corresponding to a low root-mean-square roughness of $R_\textrm{q}$ = 1.20 nm, indicating a laterally homogeneous and smooth film.

### Optical microscopy: wetting and deswelling of brushes

#### Experimental set-up

The dynamic wetting and (de)swelling of the PLMA polymer brushes by *n*-alkanes were imaged using top-view white-light optical microscopy (Fig. 1). Two microscope set-ups were used: (i) a Zeiss Axioskop 1, equipped with a 5× objective (Zeiss Plan-Neofluar 5x/0.15NA) and a Basler a2A5328-15ucBAS color camera, and (ii) a Nikon Eclipse L150 equipped with a 4x objective (CFI Plan Fluor 4x/0.13NA) and a Basler acA5472-17uc camera. The camera acquisition rate was adjusted to match the dynamics of the specific *n*-alkane under study. Early-time behavior was consistently recorded at one frame every 5 s, while slower liquids such as hexadecane were typically imaged at one frame every 30 s.

#### Measurement procedures

We demonstrate the versatility of our colorimetric approach by conducting two types of experiments to investigate two complementary dynamical processes: The first involves evaporation-driven deswelling of uniformly swollen PLMA brushes, whereas the second concerns the dynamic swelling of a dry brush following droplet deposition, where transport is governed by the coupled effects of imbibition, condensation, and evaporation.

Before each experiment, the PLMA brush is rinsed with toluene, ethanol, and deionized water and subsequently dried under a nitrogen stream. All measurements are taken in both open- and closed-cell configurations (Fig. 1a): In the open-cell configuration, the complete microscopic set-up is enclosed by a plastic cover to minimize the influence of ambient airflow. In the closed configuration, the sample is covered by a glass coverslip separated from the brush by a spacer of thickness $d \approx 0.20~\textrm{mm}$. All experiments were performed on brushes with a known dry thickness $h_0$ ranging between 155 and 170 nm.

**Fig. 3:**
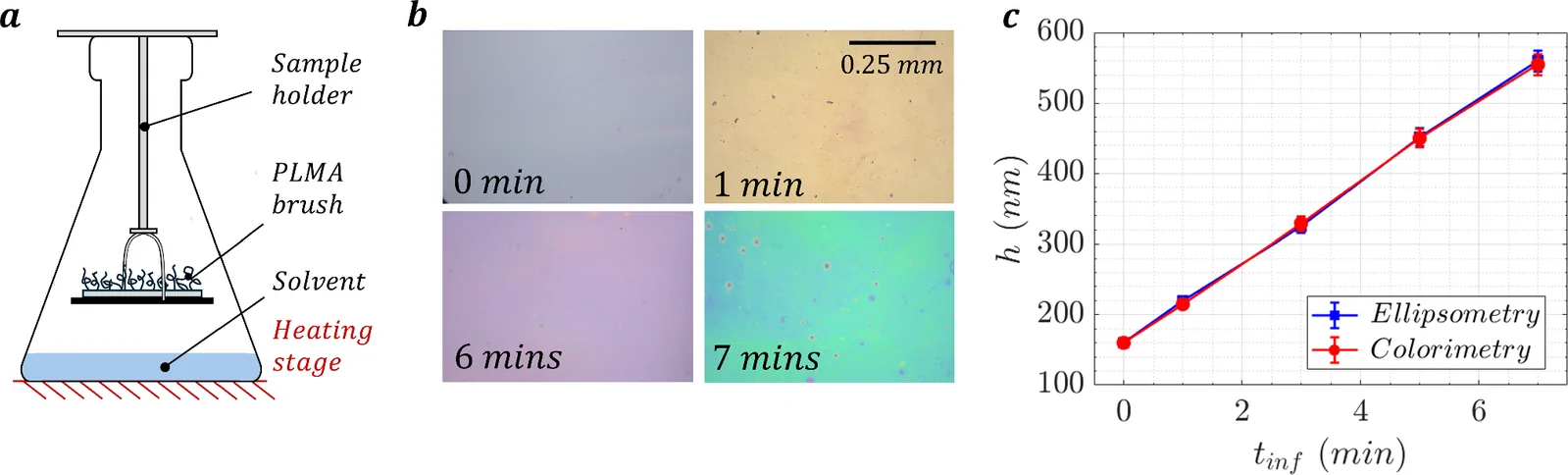
**a** Schematic of the vapor deposition set-up used to uniformly swell the brush samples with solvent. **b** Optical microscopy images of the hexadecane swollen brush samples after $t_{\textrm{inf}} = 0$-7 minutes of vapor exposure. **c** Brush thickness as a function of hexadecane infusion time, obtained from colorimetry (red) and ellipsometry (blue) for an initial dry thickness of $h_0 = 160~\textrm{nm}$

**To study evaporation-driven deswelling**, the brushes are first uniformly swollen in a vapor-enriched environment (Fig. 3a). Specifically, 20 mL of solvent is injected into a 200-mL Erlenmeyer flask and heated to $35^\circ \textrm{C}$, $57^\circ \textrm{C}$, and $75^\circ \textrm{C}$ for *n*-dodecane, *n*-tetradecane, and *n*-hexadecane, respectively. These temperatures are chosen to increase with alkane chain length, consistent with their increasing boiling points ($T_\mathrm{{b}} = 216, 253, 287 ^\circ \textrm{C}$, respectively), in order to achieve comparable vapor pressures and swelling conditions across the different solvents. The solvent is maintained at the target temperature for at least 30 min, after which dry brush samples are placed face-up on a holder and suspended at the center of the flask, sealed with parafilm (Fig. 3a).

Using this simple vapor deposition method, the initial degree of swelling, $\alpha _0 = h/h_0$, can be tuned by varying the infusion time ($t_\textrm{inf}$), enabling controlled swelling from the dry state up to the equilibrium swelling ratio $\alpha _\textrm{max}$. For all solvents the maximum swelling ratio is $\alpha _\textrm{max} \approx 4.3$. Beyond this saturation level, bulk oil forms on the top surface, indicating that the solvent chemical potential in the brush exceeds that of the free liquid ($\Delta \mu _\mathrm{{S}}^\mathrm{{mix}}>0$). All experiments were therefore performed within the swelling regime $\alpha _0 \le \alpha _\textrm{max}$, under which the swollen brushes appear laterally uniform across the field of view, as shown in the optical microscopy images (Fig. 3b). In this regime, interference colors provide a direct visual indication of the local film thickness when compared to the colormap (Fig. 5). Using the approach described in the following section (3.1), we quantify and compare the area-averaged thickness values of these samples obtained using colorimetry (Fig. 3c, red) with ellipsometric measurements (blue), showing good agreement.

The samples are then rapidly transferred to the microscope set-up, where the decrease in film thickness is monitored over time in open-cell and closed-cell conditions. Additionally, to compare evaporation rates between open-cell conditions and forced convection, the sample is fixed to the stage and exposed to a continuous nitrogen stream directed parallel to the sample surface.

Imaging continues until the swollen thickness returns to the dry brush value or until 4 h have elapsed.

**To study droplet-induced swelling**, a small *n*-alkane droplet ($V_\textrm{d} = 40-200$ nL) is deposited onto the dry brush, and the evolution of the resulting swelling gradient from the contact line is tracked. Measurements are continued until complete evaporation or for a maximum duration of 24 h.

## Quantitative colorimetry

### Mapping color to film thickness

The principle of colorimetric thickness measurements is based on the classical expression of thin-film interferometry (see, e.g., [51]). The Fresnel coefficients $r_\mathrm{{s/p}}$ describe reflection of the electromagnetic field at the interface between two dielectric media with refractive indices $n_i$ and $n_j$ as a function of the incident angle $\theta _\mathrm{{i}}$ for perpendicular (*s*) and parallel (*p*) polarization

3.1 $$\begin{aligned} r_\mathrm{{s}} = \frac{n_i \cos \theta _\textrm{i} - n_j \cos \theta _\textrm{t}}{n_i \cos \theta _\textrm{i} + n_j \cos \theta _\textrm{t}}, \quad r_\mathrm{{p}} = \frac{n_j \cos \theta _\textrm{i} - n_i \cos \theta _\textrm{t}}{n_j \cos \theta _\textrm{i} + n_i \cos \theta _\textrm{t}} \end{aligned}$$

where the transmission angle $\theta _\mathrm{{t}}$ is determined by the incident angle via Snell’s law, $n_i \sin \theta _\mathrm{{i}} = n_j \sin \theta _\mathrm{{t}}$. For a thin film of thickness *h* and refractive index $n_1$, situated on a substrate ($n_2$) in air ($n_0$), the interference between multiple internally reflected beams leads to the following effective reflection coefficient:

3.2 $$\begin{aligned} r_\mathrm{{{eff,}s/p} } = \frac{r_{01} + r_{12}e^{i\Delta \phi }}{1 + r_{01}r_{12}e^{i\Delta \phi }} \end{aligned}$$

where $r_{01}$ and $r_{12}$ are the Fresnel coefficients for the top (air-film) and bottom (film-substrate) interfaces, respectively. The term $\Delta \phi = 4\pi n_1 h \cos \theta _\mathrm{{t}} / \lambda$ represents the round-trip optical phase delay, which contains the desired information on the film thickness *h* via the optical thickness $d = n_1 h$. For common, unpolarized illumination, the total reflectivity *R* is then determined by the average of the two polarization states: $R(\lambda , \theta _\mathrm{{i}}, h) = (|r_\mathrm{{{eff},s}}|^2 + |r_\mathrm{{{eff},p}}|^2)/2$.

Everyday observations such as the shifting colors of a soap bubble or the rainbow-like sheen of an oil film on wet asphalt are direct consequences of thin-film interference. These are classical examples of qualitative colorimetry, where film thickness is inferred from the perceived color under broadband illumination. Within the range of a few tens of nanometers up to approximately $\sim 1~\mathrm {\mu m}$, an experienced observer can typically judge the film thickness *h* with an accuracy of ≈10-30 nm. However, the inference is not a direct measurement of the underlying reflectivity spectrum $R(\lambda , \theta _\mathrm{{i}}, h)$, but instead arises from the compression of spectral information into a three-channel visual response and subsequent nonlinear processing in the human visual system [52].

In contrast, modern quantitative colorimetry determines the physical thickness through a controlled optical measurement chain involving a stabilized illumination source, a microscope, and a color camera for detection. The microscope typically involves an objective with a relatively low numerical aperture (NA<0.2). In this case, the incident angle is nearly normal ($\theta _\mathrm{{i}} \approx 0^{\circ }$) for all relevant optical rays involved. Hence, the angle dependence can be neglected and the difference between *p*- and *s*-polarization vanishes, such that the total reflectivity $R(h,\lambda ,\theta _\mathrm{{i}})$ reduces to $R(h,\lambda )= |r_\textrm{eff}|^2$. The accuracy of this approximation is easily verified by comparing the expression to the full integration over the acceptance cone of the objective [53]. The illumination source is typically some form of white-light source such as a halogen lamp with a known spectral intensity distribution $I_\mathrm{{ill}}(\lambda )$. The digital camera captures the reflected light through three channels with spectral sensitivity bands $S_\mathrm{{R}}(\lambda ), S_\mathrm{{G}}(\lambda ), \text { and } S_\mathrm{{B}}(\lambda )$. These bands typically have a width of $\sim 100~\textrm{nm}$ and are centered in the blue, green, and red parts of the visible spectrum (at approximately 470, 530, and 610 nm for our specific sensor). To model the digital response, we integrate the product of the illumination, reflectivity, and sensor sensitivity over the full visible spectrum ($\lambda \in [400, 700]$ nm). The output of each channel is scaled to $0\text {--}255$ for standard 8-bit resolution (or more, for cameras with higher resolution), leading to an expected camera output of:

3.3 $$\begin{aligned} \begin{aligned}&R_{\textrm{cam}}(h) = \int I_\mathrm{{ill}}(\lambda ) R(h,\lambda ) S_R(\lambda ) \, d\lambda , \\&G_{\textrm{cam}}(h) = \int I_\mathrm{{ill}}(\lambda ) R(h,\lambda ) S_G(\lambda ) \, d\lambda , \\&B_{\textrm{cam}}(h) = \int I_\mathrm{{ill}}(\lambda ) R(h,\lambda ) S_B(\lambda ) \, d\lambda . \end{aligned} \end{aligned}$$

**Fig. 4:**
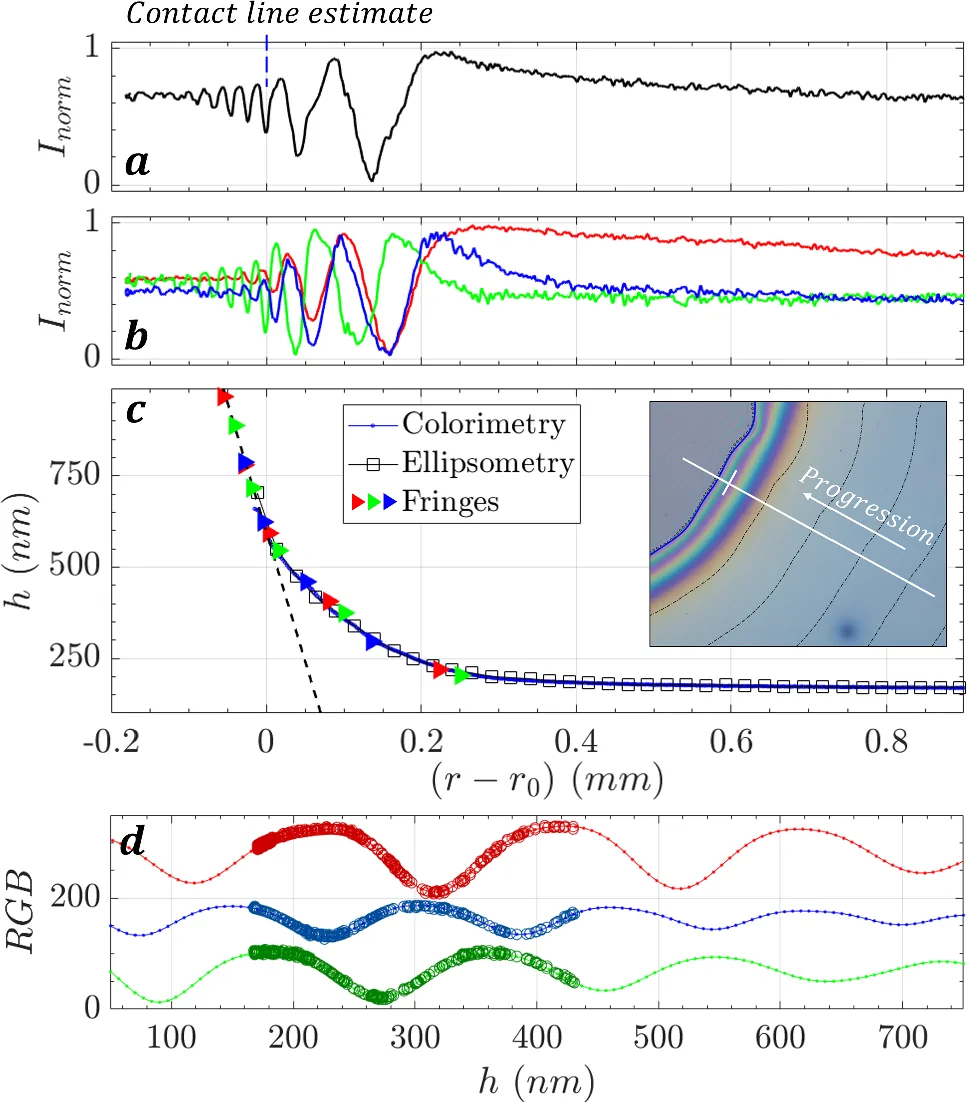
**a,b** Normalized grayscale and RGB intensity profiles measured along the outward radial normal (white progression line, panel **c** inset). **c** Film thickness *h* for an equilibrated hexadecane halo (t≈1.5 h) comparing colorimetry (blue circles), ellipsometry (black squares), and fringe counting (triangles). The dashed line shows the extrapolated linear drop profile used to estimate the contact angle and contact line position $r_0$. **d** Extracted RGB values (open symbols) closely follow the amplitude-corrected theoretical curves (in *h*-domain), with minor spread due to variances in illumination and brush morphology. The green and red channels are offset by -75,+150 for clarity

Each expected $\textrm{RGB}$ output value is thus a function of the unknown thickness *h*. In an experiment, the local thickness is then obtained at any location in an image, by a least-squares fit of the $\textrm{RGB}$ values from Eq. 3.3 to the measured ones at that specific pixel location. For the purpose of illustration, Fig. 4a shows the variation of the $\textrm{RGB}$ values along a specific line perpendicular to the macroscopic contact line; see the white line in the inset of Fig. 4c. On the right-hand side, all three signals converge to constant values corresponding to the thickness of the flat dry brush far away from the drop. Upon approaching the drop, the halo of the swollen brush gives rise to oscillations in all three channels due to interference of the dominating wavelength in each channel. Within the drop, these oscillations show constant periodicities that reflect the constant slope of the droplet surface, a characteristic from which the contact angle can be directly extracted. As one moves further into the drop, the amplitude of these oscillations gradually diminishes, a damping effect caused by the finite bandwidth of the RGB channels.

To perform the actual least-squares minimization, two additional aspects need to be taken into account: (i) in practice, the measured RGB intensities deviate slightly from the calculated values. We attribute this primarily to reflection losses in the optical path (i.e., mirrors and a beam splitter) that are not explicitly included in our estimate of the total optical transfer function. We correct this by applying a single linear scaling factor to the amplitude of each measured RGB channel to match the calculated values. The same scaling factor is then used consistently throughout the experiment. (ii) The refractive index of our swelling polymer brushes varies with the degree of swelling. We take this into account by a linear interpolation between the refractive index of pure PLMA, $n_\textrm{PLMA} = 1.47$, and the solvent $n_\mathrm{{S}}$

3.4 $$\begin{aligned} n_{1}(h) = (1-\phi ) n_\textrm{PLMA} + \phi n_\mathrm{{S}} = \frac{n_\textrm{PLMA}}{\alpha } + \frac{(\alpha -1)}{\alpha } n_\mathrm{{S}} \end{aligned}$$

Here, ϕ is the mole fraction of the solvent and $\alpha = h/h_0$ the swelling ratio. Figure 4d shows the resulting $\textrm{RGB}$ model curves (lines) as calculated from Eq. 3.3 and the measured values (open circles) extracted along the white line, plotted as a function of the brush thickness *h*. For each set of experimental $\textrm{RGB}$ values, *h* is found by minimizing the the combined residual between the calculated $\textrm{RGB}$ values:

3.5 $$\begin{aligned} VAR(h) = \sqrt{w_\mathrm{{R}} \left[ R_\mathrm{{exp}} - R_\mathrm{{cam}}(h) \right] ^2 + w_\mathrm{{G}} \left[ G_\mathrm{{exp}} - G_\mathrm{{cam}}(h) \right] ^2 + w_\mathrm{{B}} \left[ B_\mathrm{{exp}} - B_\mathrm{{cam}}(h) \right] ^2} \end{aligned}$$

where $w_\mathrm{{R/G/B}}$ are weighting coefficients (see below). Two additional considerations are worth noting: (i) as usual, the interferometric signals are periodic in *h*, and so is the variance, *VAR*(*h*). To circumvent starting at a wrong interference order, we start our analysis from a known reference thickness (typically the dry thickness far away from the drop) and restrict the minimization algorithm to a search window around the thickness of the previous pixel. (ii) The reliability of the minimization depends on how strongly the intensity of each RGB channel varies with thickness: if the intensity varies only weakly with thickness (i.e., near local extrema), different nearby values of *h* produce similar residuals, resulting in a poorly constrained minimum of *VAR*(*h*). However, because the extrema of the three RGB channels are located at different thickness values, at least one channel generally remains in a region where the intensity varies strongly with thickness (Fig. 4**d**). We introduce weighting coefficients $w_i$ in the error metric, such that channels evaluated near extrema are down-weighted ($w_i = 0$), while channels probing regions of large slope are assigned the largest weights ($w_i = 1$). In practice, we determine *h* at every pixel in a two-step process. First, an initial estimate for *h* is obtained using equal weights ($w_i = 1$). Subsequently, for each channel, the weighting coefficients $w_i$ are calculated from the distance (in thickness) between the experimental estimate and the nearest local extremum on the simulated RGB curve, normalized by the distance between that extremum and the nearest point of maximum slope on the same curve. Finally, using these weights, a recalculation of *VAR*(*h*) yields the final thickness.

This procedure results in the solid blue line in Fig. 4c, which shows the gradual transition from the flat region far away from the drop through the curved region of the partly swollen halo to the straight region of the bulk drop for $r<r_0$, where $r_0$ is the contact line of the drop. Within the resolution of the graph, the thickness profile agrees perfectly with the black open squares obtained from a measurement with the imaging ellipsometer at the same location. The maximum deviation in *h* between ellipsometry and colorimetry data amounts to $<5~\textrm{nm}$ for this dataset, with a root-mean-square deviation of $1.63~\textrm{nm}$ across the entire profile. From the profile, we can also extract the contact angle $\theta _\mathrm{{Y}}=(2.3\pm 0.1)^{\circ }$ from the slope of the straight drop surface and the position $r_0$ of the contact line. From the position where the measured surface profile deviates from the linearly extrapolated drop surface, we estimate an uncertainty of $\lesssim 3~\mathrm {\mu m}$ in $r_0$.

In contrast to imaging colorimetry, mapping ellipsometry typically acquires spatially resolved data via one-dimensional cross sections with lateral resolutions on the order of tens to hundreds of micrometers (and up to the millimeter scale for conventional scanning implementations), at acquisition rates of only 2–50 data points per minute due to sequential, multi-wavelength measurements, and time-consuming nulling procedures. By comparison, colorimetry enables the capture of full two-dimensional images at the camera’s full frame rate and resolution of the imaging optics (15 frames per second and $\Delta x = 1.87\,\mu \text {m/pixel}$ for our set-up).

**Fig. 5:**
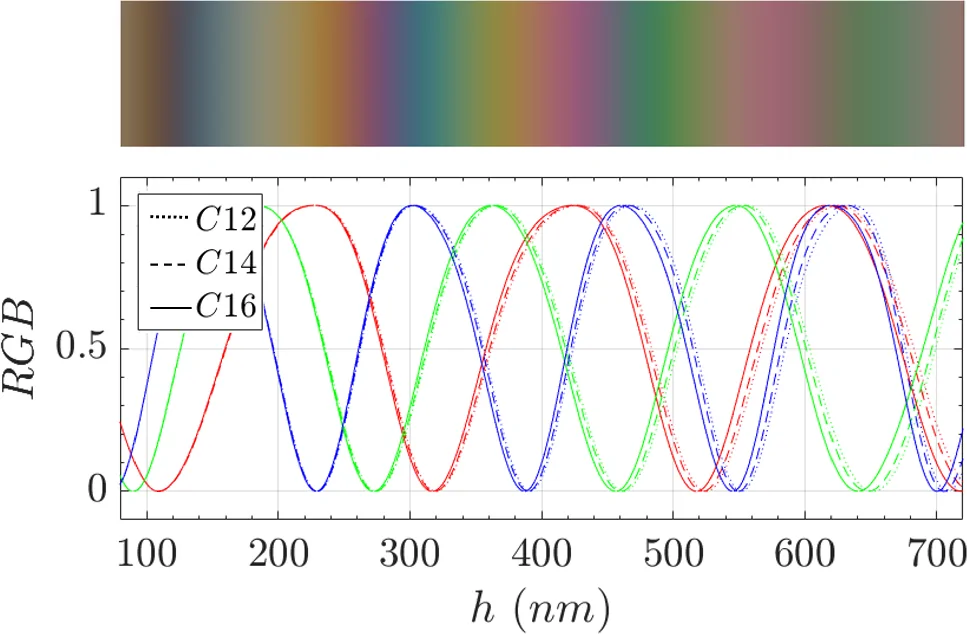
Calculated interference colormap representing the transition from film thickness *h* (*nm*) to RGB color. The model assumes an air-swollen brush-Si wafer multilayer system at near-normal incidence ($\theta _\mathrm{{i}} \approx 0^\circ$), with an initial dry brush thickness $h_0 = 175$ nm. Each color is decomposed into normalized red, green, and blue (RGB) channels, resulting in a unique mapping of intensity to film thickness. The slight shift in oscillation period reflects the varying refractive indices of the bulk liquids, as compared for dodecane (*C*12, dotted line, n≈1.42), tetradecane (*C*14, dashed line, n≈1.43), and hexadecane (*C*16, solid line, n≈1.45)

While performing experiments in the laboratory and during data analysis, it is very convenient to know how visually perceived colors translate into film thickness. To this end, we also calculate the color spectrum in the CIELAB representation (see, e.g., [52]) using the now known thickness *h* at each pixel location. LAB is a standard of three color codes defined by the CIE, the ‘Commission Internationale de l’Éclairage’ that matches the perception of the human eye much better than $\textrm{RGB}$. To this end, replace in the integrals in Eq. 3.3 the spectral sensitivity functions $S_{R/G/B}(\lambda )$ of the camera by the response functions $\bar{x}(\lambda ), ~\bar{y}(\lambda ) \text { and } \bar{z}(\lambda )$ of the three color receptors of the human eye. This leads to the so-called tri-stimulus values $X, Y, \text { and } Z$, which are subsequently transformed to the LAB color scale using another tabulated transformation. Figure 5 (top) shows the resulting color map as a function of the geometric brush thickness *h* along with the corresponding oscillating $\textrm{RGB}$ values. The increasing dispersion between the different alkanes arises from the difference in $n_1$ (Eq. 3.4) that increases with increasing swelling.

## Deswelling kinetics of PLMA brushes upon exposure to various alkanes

Having established the experimental protocols and the colorimetric data analysis procedure, we analyze two specific test cases, namely the uniform deswelling of pre-swollen brushes upon evaporation of solvent and the emergence and growth of a partially swollen halo surrounding a drop deposited onto an initially dry brush.

### Solvent evaporation from pre-swollen PLMA brushes

#### Experimental results

PLMA brushes are pre-swollen to various initial thicknesses from $\sim 250 ~\textrm{nm}$ to $\sim 750 ~\textrm{nm}$ by exposing them to vapors of dodecane, tetradecane, and hexadecane as described above (see Sect. 2.3). Subsequently, the samples are transferred to the microscope where the solvents gradually evaporate into the environment while suppressing external air flows using suitable shields surrounding sample and objective. The gradual color changes are converted into decreasing values of the brush thickness using the colorimetry procedure described in the preceding section.

**Fig. 6:**
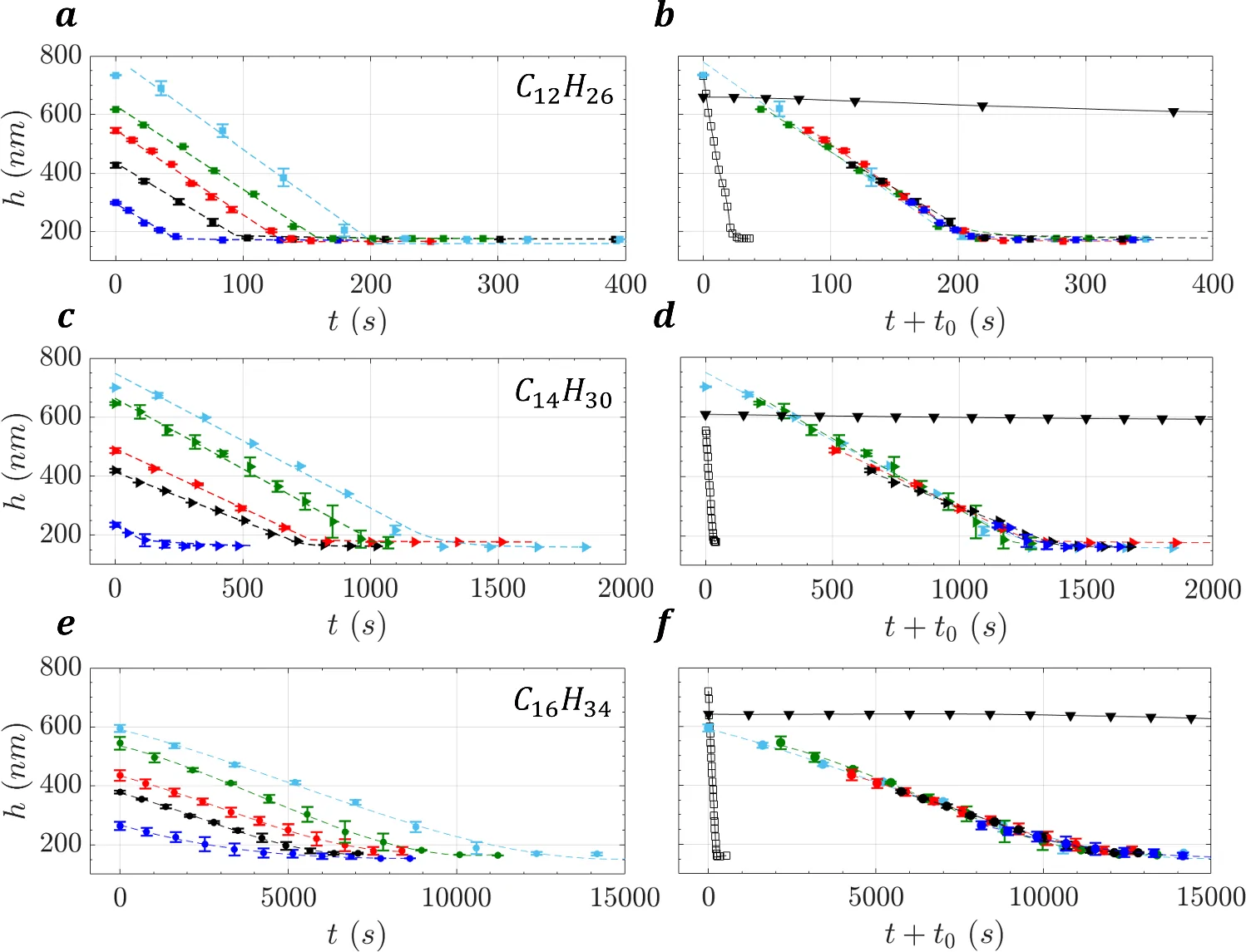
Measured decrease in thickness of uniformly swollen PLMA polymer brush layers with various initial thickness $\alpha _0$ due to evaporation in an open air environment for **a–b** dodecane, **c–d** tetradecane, and **e–f** hexadecane. The film thickness decreases linearly with time within the range $1.6 \lesssim \alpha \lesssim 4.3$. The curves collapse upon shifting the time by $t + t_0$ for all cases. In panels ${\textbf {b, d, f}}$, the closed black ▾ symbols represent evaporation in a fully closed cell, while the open black □ symbols illustrate the evaporation under an active nitrogen flow

**Fig. 7:**
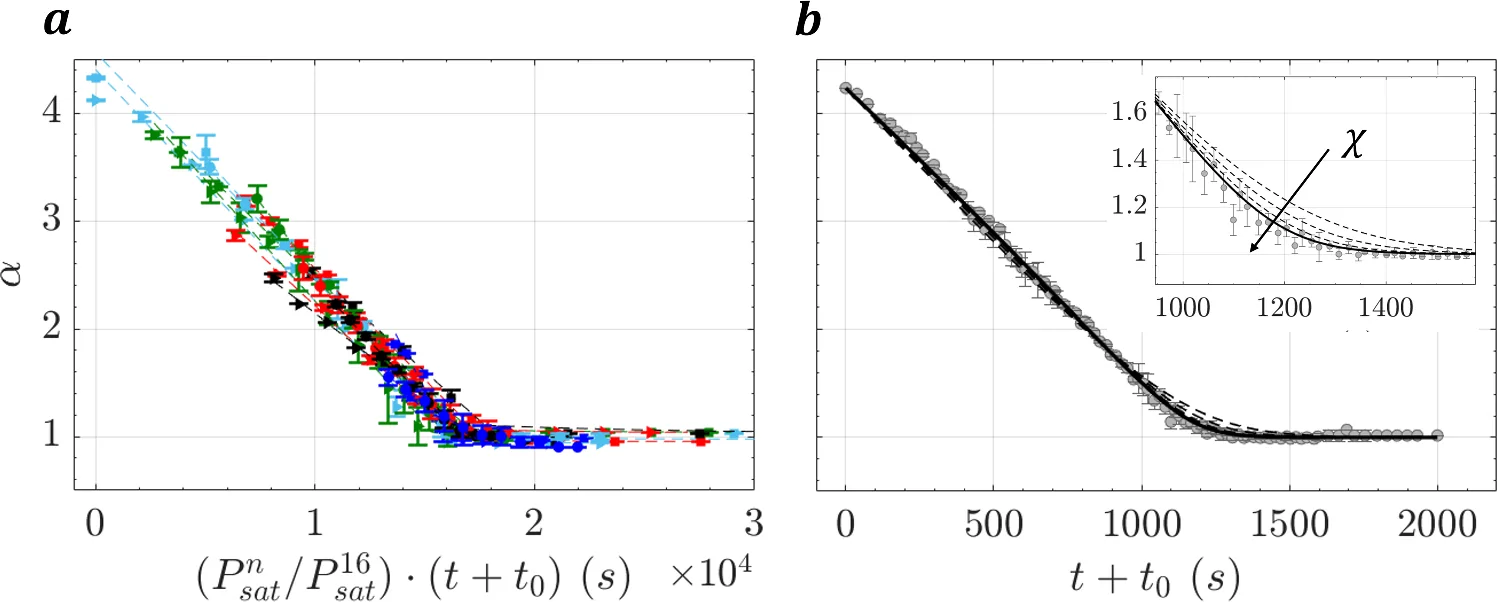
**a** Swelling ratio vs. shifted time rescaled by ratio of vapor pressures $P^n_{sat}/P^{16}_{sat}$. Squares (n=12): $C\textrm{12}$, triangles (n=14): $C\textrm{14}$, circles (n=16): $C\textrm{16}$. Data from Fig. 6. **b** Deswelling ratio vs. time from bin-averaged experimental data of $C\textrm{14}$ swollen brushes (symbols) and numerical solution of Eq. 4.5 (black line) with σ=0.05. Inset: zoomed view to final stages of evaporation with α<1.6. Model curves for increasing χ=0,0.2,0.5,0.7 (in the direction of the arrow)

For all three solvents, we find that the brush thickness decreases monotonically with time from the initial value to the final dry thickness $h_0$ (Fig. 6 left column). For each alkane, the decrease in film thickness is remarkably linear except for the final few tens of nanometers, independent of the degree of initial swelling. The latter is illustrated by the excellent overlap of all deswelling curves that can be achieved by shifting the origin of time for curves with a lower degree of initial swelling, Fig. 6 right column. Absolute de-swelling rates decrease almost two orders of magnitude from dodecane to tetradecane to hexadecane, in line with the decreasing volatility of the solvents; see Table 1. The fact that the ratio between the evaporation rate $\bar{k}$ and the saturated vapor pressure $P_\textrm{sat}$ is almost constant for the three solvents suggests that the thinning of the brushes is controlled by the diffusion of the evaporating solvent in the gas phase. This conclusion is qualitatively corroborated by the effect of a continuous air flow across the sample surface, which speeds up the process (open squares in Figs. 6b,d,f) and by placing a cover slip above the sample, which prevents evaporating solvent from escaping into the ambient environment and thereby suppresses evaporation almost completely (down triangles). More quantitatively, we can demonstrate the transport limitation by diffusion in the vapor phase by scaling time by the saturated vapor pressure. Upon doing so, all data from Fig. 6 collapse onto a single master curve, Fig. 7a.

**Table 1:** Average evaporation rate $\bar{k}$ (see SI Section 3 for complete data), saturation pressure $P_\textrm{sat}$, dynamic viscosity μ, liquid self-diffusion coefficient $D_\textrm{self}^\textrm{L}$, and vapor diffusion coefficient for dodecane, tetradecane, and hexadecane at T=298.15K [54–56]

	$\bar{k}$	$P_\textrm{sat}$	$\bar{k}/P_\textrm{sat}$	μ	$D_\textrm{self}^\textrm{L}$	$D_\textrm{v}$
Solvent	[$1/\text {min}$]	[$\text {Pa}$]	[$1/(\text {min}\cdot \text {Pa})$]	[$\text {mPa}\cdot \text {s}$]	[$10^{-10}~\text {m}^2/\text {s}$]	[$10^{-6}~\text {m}^2/\text {s}$]
*n*-C$_{12}$H$_{26}$	193.34 ± 13.03	17.87	10.82 ± 0.73	1.35	8.14	5.10
*n*-C$_{14}$H$_{30}$	24.41 ± 3.43	1.86	13.10 ± 1.84	2.07	5.20	4.50
*n*-C$_{16}$H$_{34}$	2.13 ± 0.43	0.20	10.70 ± 2.16	3.06	3.76	4.00

Note how the ratio $\bar{k}/P_\textrm{sat}$ is approximately constant

#### Modeling of gas phase-limited evaporation kinetics

To analyze the deswelling rate quantitatively, we consider a simple one-dimensional model that is controlled by diffusion in the gas phase. Thereby, we neglect possible interfacial transfer resistances that were considered in earlier gradient dynamics models [36, 38]. In this case, the solvent flux in the gas phase in steady state is given by:

4.1 $$\begin{aligned} j_{\textrm{v}} = -\frac{D_{\textrm{v}}}{L} (c_{\textrm{S}} - c_L) \approx -\frac{D_{\textrm{v}}}{L} \frac{P_{\textrm{S}}}{kT} \end{aligned}$$

where $D_\mathrm{{v}}$ is the diffusion coefficient of solvent in air, $c_\mathrm{{S}}$ is the vapor concentration (particles/volume) at the brush surface and $c_{{L}} = 0$ the far-field concentration at a distance *L*. On the right-hand side, we inserted the ideal gas law to express $c_\mathrm{{S}}$ in terms of the solvent pressure $P_\mathrm{{S}}$ at the surface. Equilibrium at the brush surface implies that the chemical potentials of the solvent in the vapor phase $\mu _\mathrm{{v}}^\mathrm{{S}}$ and in the brush $\mu _\mathrm{{b}}^\textrm{S}$ are equal. Using standard ideal gas expressions, the former is given by:

4.2 $$\begin{aligned} \mu _\mathrm{{v}}^\mathrm{{S}} = \mu _\mathrm{{v}}^\mathrm{{sat}}(T) + kT \ln \left( \frac{P_\mathrm{{S}}}{P_\textrm{sat}} \right) \end{aligned}$$

For the polymer brush, we express the solvent chemical potential as a function of the solvent volume fraction ϕ following Birshtein [44, 57], where a uniform polymer density profile is assumed, as originally proposed in the Alexander–de Gennes model [58, 59]. Rewriting this expression in terms of the measured swelling ratio $\alpha = h/h_0$, the excess swelling ratio reads

4.3 $$\begin{aligned} \Delta \tilde{\mu }_\mathrm{{b}} =\frac{\mu _\mathrm{{b}}^\mathrm{{S}} - \mu _\mathrm{{b}}^\mathrm{{sat}}(T)}{kT} = \chi \alpha ^{-2} + \ln \left( \frac{\alpha - 1}{\alpha } \right) + \alpha ^{-1} + 3 \sigma ^2 \alpha \nonumber \\ \end{aligned}$$

where χ represents the Flory–Huggins interaction parameter and σ the dimensionless grafting density. The reference value $\mu _\mathrm{{b}}^\mathrm{{sat}}(T)$ is chosen such that $\Delta \tilde{\mu }_\mathrm{{b}}$ vanishes at maximum swelling $\alpha _\textrm{max}$. Equating the chemical potentials and substituting the result into Eq. 4.1 yields:

4.4 $$\begin{aligned} j_\mathrm{{v}} =\frac{D_\mathrm{{v}} P_\textrm{sat}}{kT L} e^{\Delta \tilde{\mu }_\textrm{b}(\alpha )} \end{aligned}$$

Assuming a vertically averaged (constant) number density $\rho _\textrm{S}$ of solvent in the brush, the flux of evaporating solvent $j_\mathrm{{b}}$ is directly proportional the reduction of brush thickness as $j_\mathrm{{b}} =-\rho _\textrm{S}\dot{h}=-\rho _\textrm{S}h_0\dot{\alpha }$. Balancing the two fluxes at the interface finally yields the differential equation

4.5 $$\begin{aligned} \dot{\alpha } = -K e^{\Delta \tilde{\mu }_\mathrm{{b}}(\alpha )} \end{aligned}$$

where $K=D_\mathrm{{v}}P_\textrm{sat} / kTL\rho _\mathrm{{{\ell }}}h_0$ is a kinetic prefactor that serves as an adjustable parameter. Figure 7b shows a comparison between a representative evaporation curve ($C_{14}$) and the numerical integral of Eq. 4.5 for an initially maximally swollen brush with α(0)=4.25. Inserting the known values for all other parameters (see Table 1), the fitted value for *K* yields $L=2-4\textrm{mm}$, consistent with the expected macroscopic distance over which these solvent diffuse into the ambient environment. Data for other alkanes are found in the Supporting Information, SI 3.2 Fig. S4 and in Fig. 7b.

**Fig. 8:**
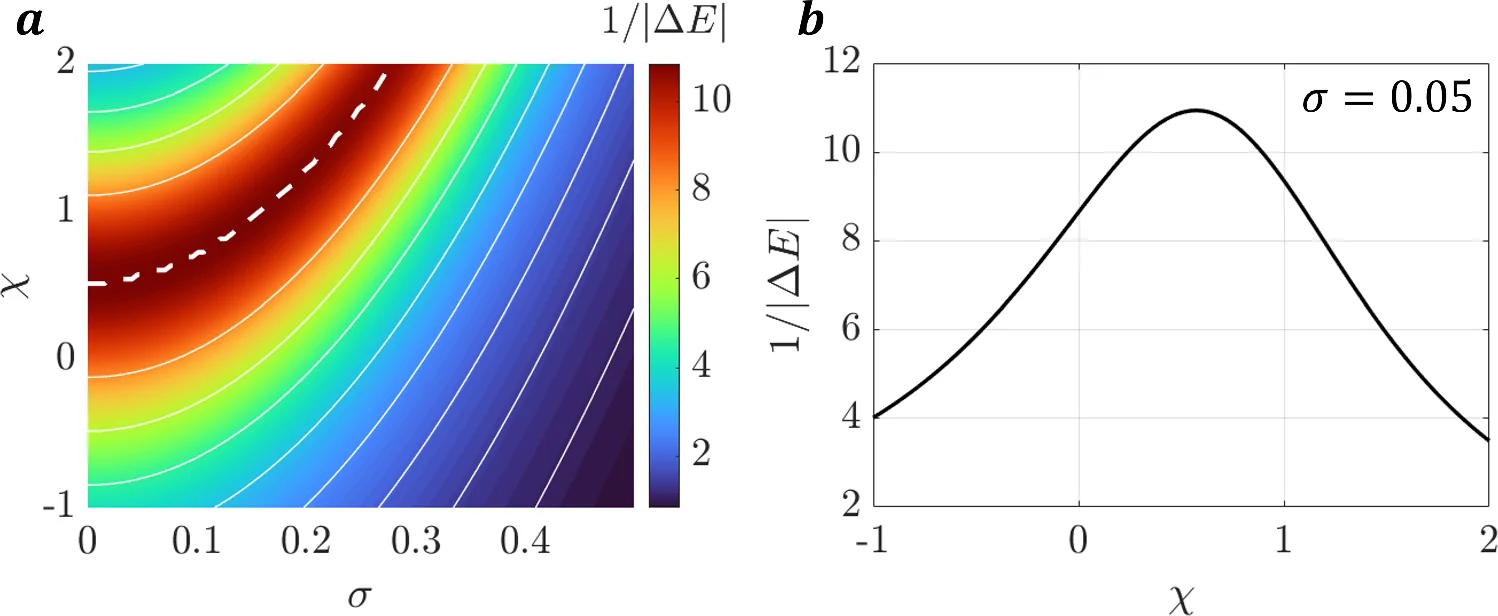
**a** Map of the fit quality across the $\sigma /\chi$ parameter space, where the color scale shows the inverse residual error (1/|ΔE|) between the experimental data (*C*16) and calculated de-swelling trends. The dashed white line traces the combination of parameters for which the discrepancy between experiment and theory is minimized. **b** Residual error as a function of χ for a vertical cross section of the parameter space at σ=0.05

The model reproduces all salient features of the experimental observation: (i) the brush thickness decreases linearly with time for values over $1.6 \lesssim \alpha \lesssim 4.2$. Physically, this linear decrease arises from the fact that $\Delta \tilde{\mu }_\mathrm{{b}}\ll 1$ within this range according to Eq. 4.3, which implies $P_\textrm{S}\approx P_\textrm{sat}$. (ii) Once α decreases below ∼1.6, the logarithmic term in Eq. 4.3 leads to a strong decrease in $\Delta \tilde{\mu }_\mathrm{{b}}$ reflecting the entropically driven retention of solvent within the brush. This process is responsible for the deviation from the linear behavior for small α and hence for the gradual slowing down of the evaporation process. More broadly, the transition from a linear evaporation regime at high swelling ratios to a slower regime at lower swelling ratios may provide a useful framework for interpreting recent reports of transient evaporation kinetics upon exposing brushes to vapors of variable degrees of solvent saturation [60, 61].

Interestingly, the transition from the linear evaporation regime to the final dry state reveals even more information about the solvent-polymer interaction. As shown in the inset of Fig. 7b, the curvature in this transition region depends sensitively on the value of χ. For the present dataset (*C*14), an optimum fit is found for $\chi \approx 0.7$, while χ=0 is incompatible with the experimental data. Such a high value may seem surprising in view of the fact that our PLMA brushes swell rather well in the presence of alkanes, as evidenced by the values of $\alpha _\textrm{max} \approx 4.3$ (Sect. 2.3). Note, however, that the deviation is only observed in the range of α<1.6. Recent experiments [62] as well as numerical calculations in this special issue [63] suggest that χ varies with the degree of swelling in various brush systems and indeed increases with decreasing α. To analyze the consequences of this effect, the constant χ parameter needs to be replaced by a swelling-dependent interaction function, which changes the expression for $\Delta \tilde{\mu }_\textrm{b}$ in Eq. 4.3. Such an analysis is beyond the scope of the present work and will be discussed elsewhere in the context of additional experimental data [64].

Furthermore, the dimensionless grafting density estimated as $\sigma \approx 0.05$ (see SI Section 1) modulates the contribution of chain stretching to the chemical potential and is found to correctly reproduce the linear regime for $1.6 \lesssim \alpha \lesssim 4.2$. Increasing σ values beyond 0.2 amplifies the elastic stretching term in Eq. 4.3, causing the predicted evaporation profile to deviate from linearity and develop a pronounced curvature that cannot be compensated for by adjusting χ within physically reasonable limits, as shown in SI 3.3, Fig. S5.

The combined influence of the interaction parameter χ and the grafting density σ constrains the range of possible $(\sigma , \chi )$ combinations that minimize the discrepancy between theoretical predictions and experimental data. To quantify this, we evaluate the fit quality over a broad parameter space ($0 \le \sigma \le 0.5$ and $-1.0 \le \chi \le 2.0$, Fig. 8a) for hexadecane, where the color scale indicates the inverse residual error (1/|ΔE|). The dashed white line traces the parameter pairs resulting in the best agreement, whereas the blue regions correspond to poor fits. Additional physical considerations further restrict the plausible parameter range: values of σ>0.2 correspond to excessively dense brushes with limited conformational freedom, while values of χ>1.0 are generally unrealistic because they imply very poor brush-solvent miscibility.

Taking our estimate σ=0.05 as an example, Fig. 8b shows the residual error distribution as a function of χ for a vertical cross section at fixed grafting density, with a maximum at χ=0.575. Although the relatively broad full width at half maximum of this peak indicates that a range of χ values remains plausible for a given σ, this uncertainty can be reduced by fitting individual swelling curves rather than bin-averaged experimental datasets. The details of the evaporation curves thus offer a convenient approach to constrain χ and σ, i.e., two crucial parameters for the modeling of any swelling polymer brush.

### Halo development for different *n*-alkanes

#### Experimental thickness profiles

The second test case demonstrating the versatility of the colorimetric approach involves the analysis of dynamic halo profiles that form ahead of *n*-alkane droplets. This phenomenon was first characterized in the context of polymer brushes in earlier work [39], based on the more general framework of solvent-induced swelling and precursor-front propagation in soft elastic materials described by Lequeux et al. [65]. When a hexadecane droplet contacts a dry brush layer, solvent begins to imbibe into the polymer network, causing the network to swell. The resulting radial solvent gradient propagates outward from the contact line, driving the expansion of a halo. In open air, the halo reaches a finite width due to a balance of imbibition and evaporation similar to the spreading of volatile fluids in porous media [66]. If evaporation is suppressed by placing a cover slip a few micrometers above the sample, the halo continues to expand throughout the sample over the course of a few days. However, a detailed analysis of the competition between imbibition and vapor-phase transport was hampered by the lack of experimental data across a sufficiently wide-range conditions to constrain the multitude of parameters in numerical models [38, 39].

**Fig. 9:**
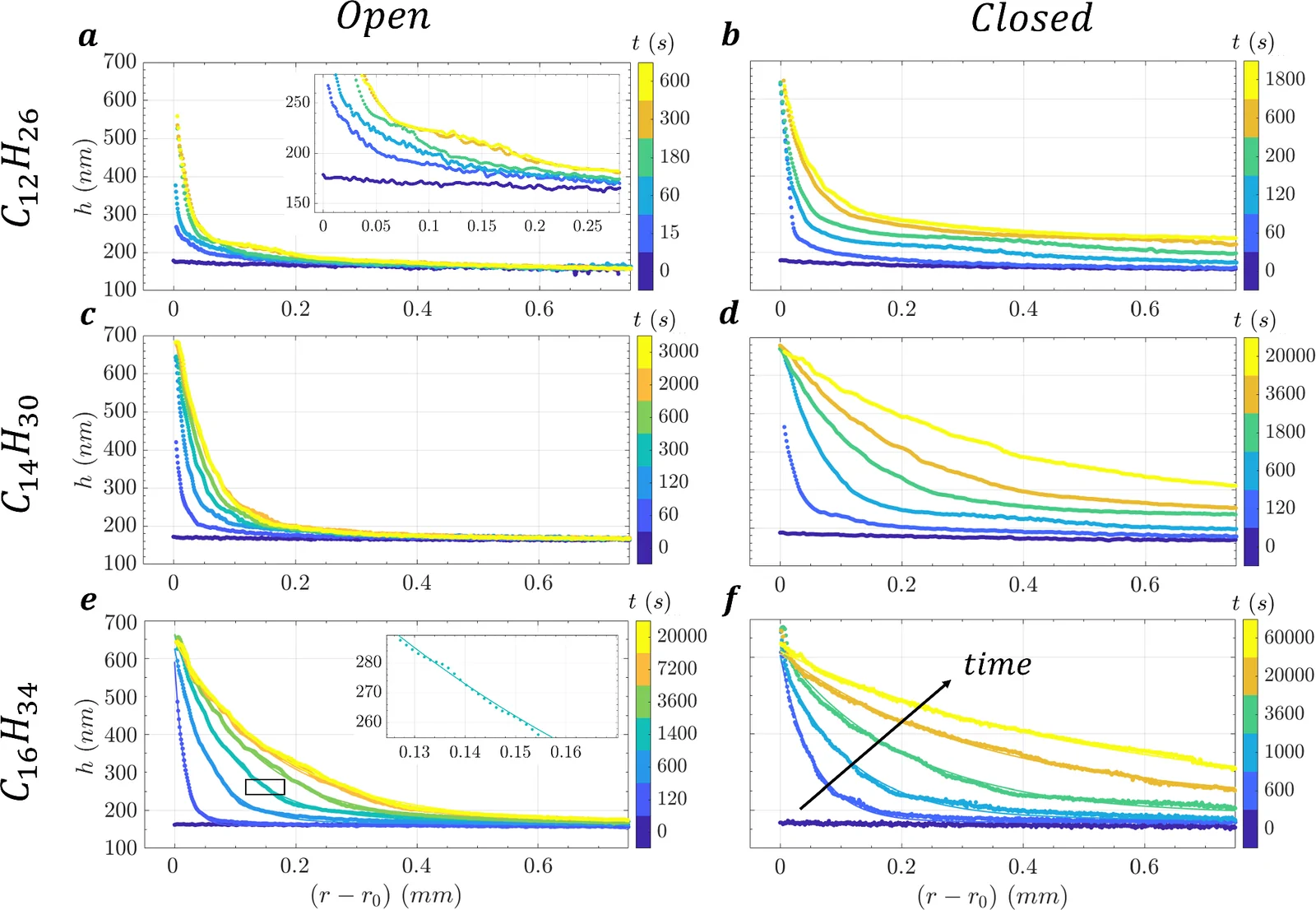
Spatio-temporal evolution of the swelling profiles h(t,Δr) extracted via colorimetry near the droplet contact line ($\Delta r = r - r_0$). The progression toward equilibrium is shown for **a–b** dodecane, **c–d** tetradecane, and **e–f** hexadecane. Profiles are compared for an open-cell configuration (left column) and a closed-cell configuration (right column). The temporal evolution, indicated by the color gradient, shows significantly different timescales across the three solvents. The inset in panel **a** shows a small stretching foot that forms ahead of the main swelling front. In panels **e–f)**, solid lines represent exponential fits that show excellent agreement with the experimental data (inset **e**)

With the increased spatial and temporal resolution offered by our colorimetric approach, we now extend the previous investigations on halo development to multiple *n*-alkanes, by placing drops of dodecane (Fig. 9a,b), tetradecane (Fig. 9c,d), and hexadecane (Fig. 9e,f) onto (identical) dry brush samples in open (left column) and closed-cell (right column) configuration. Note that, while all profiles in Fig. 9 are plotted on the same spatial scale, the timescale varies by two orders of magnitude between the different panels, as indicated by the color bar (this variation in timescale is similar to the one in the evaporation experiments discussed in the preceding section).

In open air (left column), the halo profiles eventually reach a steady state for all solvents (yellow). These steady-state configurations are characterized by a fully swollen brush at the contact line ($\Delta r = r - r_0$), which gradually decreases in thickness toward the dry brush far away. The time required to reach this steady state depends significantly on the solvent, ranging from $\approx 300~\text {s}$ for dodecane to $\approx 1.5~\text {h}$ for hexadecane. Despite these large differences in timescales, two key features emerge: (i) the profiles retain a remarkably similar shape throughout their evolution and (ii) the final halo profile extends significantly further for hexadecane than for the shorter-chain alkanes. As we shall see in the following, the latter is caused by the difference in evaporation rates.

In contrast, the halo profiles measured in the closed-cell configuration (right column) extend further and develop more rapidly. This effect is most pronounced for the shorter alkanes, where vapor accumulation reduces the net evaporation rate through enhanced re-condensation, in particular far away from the drop. In the ideal limit of an infinite solvent reservoir, equilibrium in a closed cell would correspond to a uniformly swollen state at a swelling ratio $\alpha _\textrm{max} \approx 4.3$ (see Sect. 2.3). In practice, the droplet volume is finite, and even in a closed-cell configuration the droplet slowly evaporates over long times. As the droplet shrinks and the contact line recedes, the halo profile relaxes sufficiently fast such that its shape, when viewed in the frame of the moving contact line, remains essentially unchanged.

A more detailed comparison between open- and closed-cell configurations reveals that the early-time evolution (t<3600s, i.e., within the first hour) of the hexadecane halo is nearly identical in both cases. Aside from minor, spatially uniform swelling in the far field caused by vapor condensation, halo growth is driven almost exclusively by liquid-phase imbibition. In contrast, for dodecane and tetradecane, the difference in instantaneous halo volume $V_\textrm{S}(t)$ between the two configurations is far greater. In the open air, their halo volume remains smaller than that of hexadecane, whereas in the closed cell, it becomes substantially larger due to enhanced imbibition and condensation rates, giving rise to the observed mirrored pattern in halo volumes (SI. 4, Fig. S7).

To quantitatively understand the variations in observed halo width, we consider a global mass balance of the halo ($r \in [r_0, R(t)]$, Fig. 1b). The change in the number of solvent molecules *N* in the halo region arises from the imbalance between total in- and outflow and from the change in halo size as the boundary moves at a velocity $\dot{R}$ (see e.g., [67]):

4.6 $$\begin{aligned} \frac{dN}{dt} = J_\textrm{in}-J_\textrm{out} + 2\pi \rho _\textrm{S} h(R,t) R \dot{R} \end{aligned}$$

Diffusive feeding from the drop occurs at a rate $J_\mathrm{{in}} = 2 \pi \rho _\textrm{S} r_0 q(r_0,t) = -2\pi r_0 h(r_0) D_\textrm{eff} \partial _r c|_{r=r_0}$ and varies only weakly in time due to the slow relaxation of the swelling gradient $\partial _r c$. In contrast, as shown in the preceding section, the solvent evaporation flux ($j_\mathrm{{v}}$) is essentially independent of the degree of swelling. Therefore, the total evaporative flux $J_\textrm{out} = 2\pi \int _{r_0}^{R(t)} r j_\textrm{v}(r,t) \ dr$ scales with the vapor pressure $P_\textrm{sat}$ and, in particular, with the width of the halo. A steady state is reached when this nearly constant influx balances the size-dependent evaporative loss, such that dN/dt=0 and the front no longer advances $\dot{R} = 0$. Balancing the diffusive influx and evaporative outflow results in a characteristic length scale for the final halo width $R_{\star }$:

4.7 $$\begin{aligned} R_{\star } \sim \left( \frac{2 r_0 {h(r_0)} D_{\textrm{eff}} (\partial _r c)_{r_0}}{K_0 e^{\Delta \tilde{\mu _\textrm{b}}}} \right) ^{1/2}, \quad K_0 = \frac{D_\textrm{v} P_\textrm{sat}}{kTL} \nonumber \\ \end{aligned}$$

The relation explicitly shows how the halo width is determined by the ratio of lateral solvent transport within the brush ($D_\textrm{eff}$) to vapor-phase removal ($D_\textrm{v}, P_\textrm{sat}$), leading to a square-root scaling characteristic of diffusive–evaporative processes, as discussed previously by Seker et al. [66] for the imbibition of volatile fluids into porous films.

**Fig. 10:**
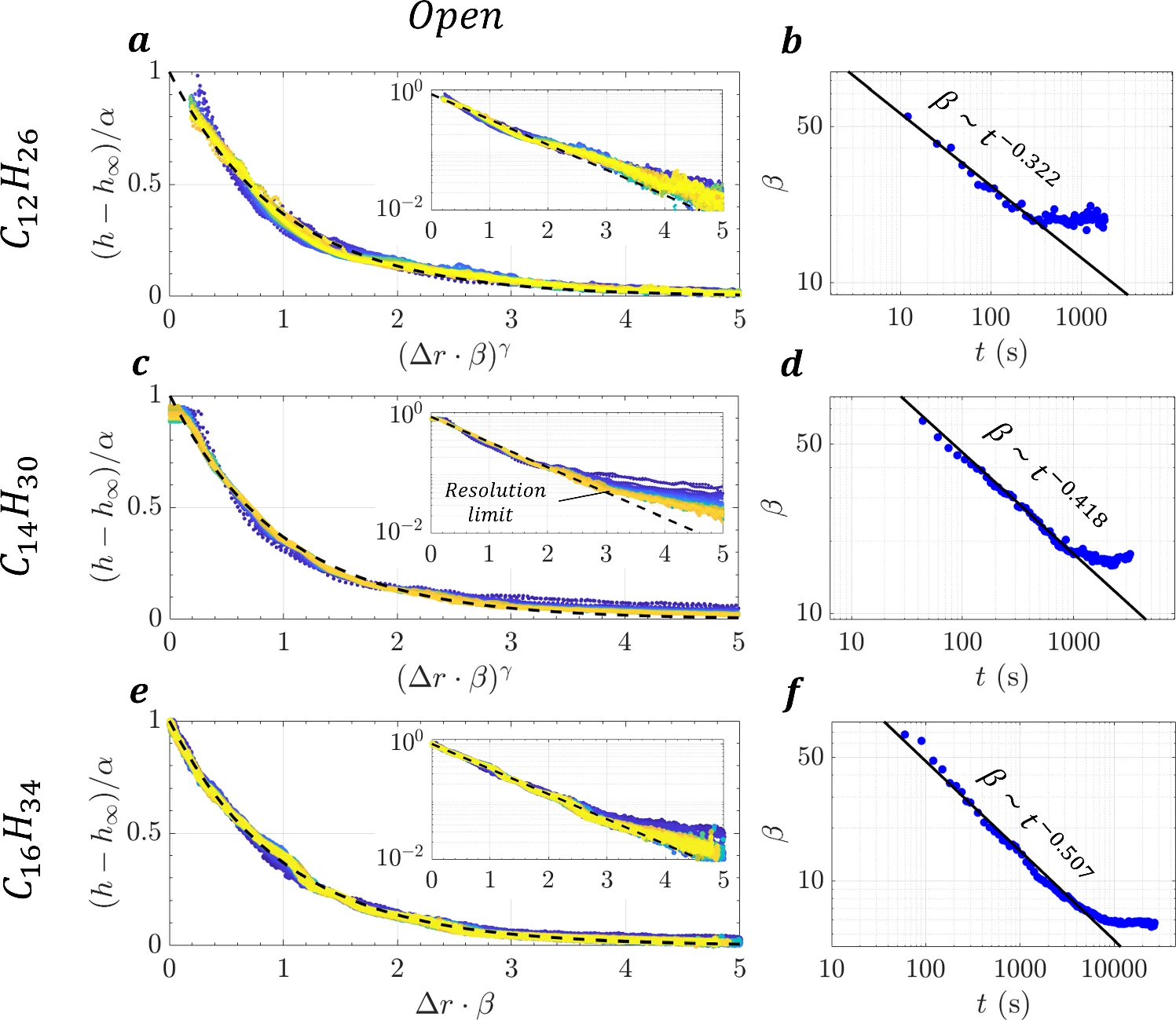
Collapse of the swelling profiles across several decades (all experimental profiles) for an open-cell configuration. The lateral distance is scaled as $(\Delta r \cdot \beta )^{\gamma }$ and the normalized height as $(h - h_{\infty })/\alpha$. **e** Hexadecane profiles exhibit a pure exponential collapse with γ=1, whereas **c** tetradecane and **a** dodecane follow a stretched-exponential behavior with γ=0.98 and γ=0.76, respectively. Insets show the collapse on a semi-logarithmic scale. Panels **b, d, f** show the decay length β(t) as a function of time for dodecane, tetradecane, and hexadecane, respectively

The observed variations in steady-state halo width across different solvents follow directly from this balance. While shorter-chain *n*-alkanes diffuse somewhat faster through the brush than longer-chain molecules, this increase in transport rate is modest, with the bulk self-diffusion ratio $D_\textrm{self}^{C{12}}/D_\textrm{self}^{C{16}} \approx 2.16$ (Tab. 1), providing an upper-bound estimate for the effective diffusion coefficients in the brush. In contrast, vapor-phase transport depends much more strongly on chain length, with rates differing by nearly two orders of magnitude ${\bar{k}^{C{12}}}/{\bar{k}^{C{16}}} \approx 91$ (Sect. 4.1). Consequently, following Eq. 4.7, the balance between lateral influx and evaporative loss leads to larger halo radii for less volatile solvents, i.e., $R_{\star }^{C12}< R_{\star }^{C14} <R_{\star }^{C16}$.

Finally, we estimate the effective lateral transport coefficient $D_\textrm{eff}$ from the experimentally measured steady-state swelling profiles of the open-cell configuration (Fig. 9). The halo region is defined by a cutoff radius $R_\star$, taken as the radial position where the brush thickness approaches its dry value; in practice, we use $h \approx 200\,\textrm{nm}$. Within this region, the concentration gradient at the droplet edge is obtained from fits to the thickness profiles as $(\partial _r c)_{r_0} = (\rho _\textrm{S}/(\alpha ^2 h_0))\, (\partial _r h)_{r_0}$. The total evaporative loss $J_\textrm{out}$ is computed by integrating the local evaporation flux $j_v = K_0 a(\alpha )$ over the halo region ($r \in [r_0, R_\star ]$), while the length scale *L* entering the evaporation prefactor $K_0$ is obtained from the evaporation analysis in Sect. 4.1 (Tables 1 and S7).

Balancing the inflow and evaporative outflow yields the bulk-averaged values $D_\textrm{eff} = 3.91,\, 2.36,\, 1.57 \times 10^{-11} ~\mathrm {m^2/s}$ for *C*12, *C*14, and *C*16, respectively. These values are approximately one order of magnitude smaller than the corresponding bulk self-diffusion coefficients (Table 1), consistent with the reduced mobility of solvent molecules due to confinement within the polymer brush, and decrease systematically with increasing chain length.

### Collapsing film profiles

Motivated by the observation that the overall shapes of the swelling profiles remain remarkably similar over time, we attempt to identify a universal halo profile. As it turns out, the swelling profiles in the open-cell configuration can all be described by a stretched-exponential function of the form:

4.8 $$\begin{aligned} h(t, r) = h_{\infty }(t) + \alpha (t) \exp [ - \beta (t) (r - r_0)^{\gamma }] \end{aligned}$$

where $h_\infty (t)$ is the far-field brush thickness, α(t) and β(t) are time-dependent coefficients, while γ is a constant. For hexadecane, the bold lines in Fig. 9e,f show these approximations, which reproduce the swelling profiles very well. When the dimensionless height $(h - h_\infty )/\alpha$ is plotted against the dimensionless distance $(\beta \cdot (r-r_0))^{\gamma }$, all curves for dodecane (Fig. 10a), tetradecane (Fig. 10c), and hexadecane (Fig. 10e) collapse onto single master curves per solvent. For hexadecane, more than 200 profiles collapse exponentially over four decades in time with a stretching exponent γ=1. In contrast, the profiles for tetradecane and dodecane follow stretched-exponential forms with γ=0.98 and γ=0.76, respectively.

In the semi-logarithmic representations (insets Fig. 10a,c,e), the profiles are shown to follow the stretched-exponential forms over several characteristic lengths (>3ℓ(t) with $\ell (t) = 1/\beta (t)$) before bending away in the far field. The deviation originates from the vertical resolution limit of the colorimetric method; once the halo approaches the dry thickness, the remaining height variations fall below resolvable thickness (≈2.0 nm). As the halo broadens over time, an increasingly larger portion of the exponential (or stretched-exponential) tail enters the resolvable range, which is reflected by the color gradient from blue to yellow, progressively approaching the theoretical curves (black dashed lines).

The collapse of the swelling profiles onto fixed spatial forms *F* implies that the time evolution can be factorized into a time-dependent amplitude α(t) and a single lateral scale $\beta (t) = 1/\ell (t)$ (right column), such that $h(r,t) \sim \alpha (t)F(\beta (t)\Delta r)$. For hexadecane (Fig. 10f), the decay parameter follows $\beta (t) \propto t^{-1/2}$ (equivalently $\ell (t) \sim \sqrt{t}$), reminiscent of the classical Washburn dynamics observed in diffusion-limited or capillary-driven spreading [68]. Together with the purely exponential profile collapse, this behavior points to a single rate-limiting mechanism controlling lateral transport over the full temporal evolution.

Therefore, for hexadecane, the collapse into a fixed, purely exponential shape requires a strong separation of intrinsic timescales: $\tau _z \ll \tau _r \ll \tau _{\text {evol}}$, where $\tau _z$ is the vertical relaxation time within the brush, $\tau _r$ the lateral transport time, and $\tau _{\text {evol}} = \ell /\dot{\ell }$ the slow evolution of the halo extent. Given the ratio $\tau _z/\tau _r \sim (H/L)^2 \ll 1$, the brush maintains vertical equilibrium throughout the experiment. Consequently, on the intermediate timescale $\tau _r$, the profile near the contact line rapidly reaches a quasi-steady balance, while the overall amplitude α(t) and lateral scale β(t) evolve only gradually on the longer, evaporation-, and imbibition-controlled timescale $\tau _{\text {evol}}$. Under these conditions, the dynamics reduce to an effectively one-dimensional radial transport problem characterized by a single evolving length scale $\ell (t) = 1/\beta (t)$, naturally resulting in the observed profile collapse.

In contrast, shorter-chain solvents exhibit stretched-exponential profiles where β(t) follows sub-diffusive scaling, with exponents below <1/2 (0.42 and 0.33 for tetradecane and dodecane, respectively, Fig. 10d,e). This behavior is reminiscent of spreading observed in systems affected by substrate heterogeneity, viscoelastic dissipation, and nonlinear mobilities [69, 70] (while the scaling exponent decreases for shorter chains, the absolute rate of halo growth remains higher due to larger prefactors, reflecting their lower effective resistance to flow i.e., reduced bulk viscosity and related dissipative contributions). Stretched-exponential forms and sub-diffusive dynamics may emerge from several factors, including i) the interplay of multiple dominant transport mechanisms, ii) a broad distribution of relaxation timescales, and iii) a solvent-dependent mobility [64]. A detailed analysis of these processes is beyond the scope of the present analysis.

## Conclusion

In this work, we establish quantitative colorimetric interferometry as an effective approach to resolve the spatiotemporal evolution of swelling in polymer brush systems. By combining nanometer-scale vertical sensitivity with high lateral resolution and real-time image acquisition, we have overcome key limitations of existing techniques, enabling direct measurement of transient thickness fields over relevant length and timescales. While demonstrated here for drops of alkanes on PLMA brushes, the approach is broadly applicable to thin soft matter films, offering new opportunities to study coupled transport and material response in wetting phenomena.

Beyond the methodological advance, our experiments provide direct insights into the mechanisms governing solvent transport by systematically varying vapor pressure over nearly two orders of magnitude. For the *n*-alkanes studied (C$_{12}$H$_{26}$, C$_{14}$H$_{30}$, C$_{16}$H$_{34}$), we show that evaporation from pre-swollen brushes is limited by vapor-phase transport, rather than by vertical transport within the brush or transfer across the interface. The collapse of the deswelling profiles when rescaled by vapor pressure, together with the excellent agreement between the experimental data and our simple diffusion model, strongly supports this interpretation and places constrains on key transport parameters in theoretical models [36, 38, 39]. The dominance of vapor-phase transport sets bounds on effective mobilities and enables evaporation experiments to be used as a sensitive probe for estimating thermodynamic parameters, such as the Flory–Huggins interaction parameter and the grafting density. This opens a route to connect evaporation kinetics to equilibrium brush thermodynamics, although more refined analysis will be needed to extract concentration-dependent interactions.

From the droplet-induced swelling experiments, our results confirm that halo formation arises from the competition between imbibition and evaporation–condensation processes. The final halo width is determined by the balance between the near-constant diffusive influx and the size-dependent evaporative outflux. Furthermore, the temporal profiles collapse onto similar functional forms that are well described by exponential and stretched-exponential behavior. For hexadecane, the exponential collapse suggests a single mechanism governing the lateral spreading of the halo, with a decay length reminiscent of classical Washburn spreading. For the shorter-chain solvents, we observe stretched-exponential profiles exhibiting sub-diffusive spreading reminiscent of transport in systems influenced by substrate heterogeneity. Together with the evaporation results, these findings provide a deeper understanding of the transport properties that regulate polymer brush swelling

Overall, our results demonstrate that time-resolved, full-field thickness measurements provide both improved experimental access and a means to quantitatively constrain models of adaptive soft interfaces. We expect this approach to be broadly applicable to responsive systems in which coupled transport and structural evolution determine functionality and to enable systematic extraction of material parameters from dynamic experiments.

## Supplementary Information

Below is the link to the electronic supplementary material.

Supplementary file 1 (pdf 4376 KB)

## Data Availability

The experimental data and analysis code are available from the corresponding author upon reasonable request.
