# Supplementary material for: Characterization of swelling gradients and evaporation dynamics in polymer brushes using high-resolution colorimetry
Source: Eur Phys J E Soft Matter. 2026 Jul 30;49(8):67. doi: 10.1140/epje/s10189-026-00613-8 (PMC13423925; doi:10.1140/epje/s10189-026-00613-8)
Supplement: Supplementary file 1 — (pdf 4376 KB) [file 10189_2026_613_MOESM1_ESM.pdf]

Supporting information: Characterization of swelling gradients and evaporation dynamics in polymer brushes using high-resolution colorimetry

Vincent Siekman[1†], Sander Reuvekamp[1,2†], Enqing Liu[1], Sissi de Beer[2], Frieder Mugele[1*]

[1]Department of Chemical Engineering, University of Twente, Drienerlolaan 5, Enschede, 7522 NB, The Netherlands.
[2]Department of Molecules & Materials, University of Twente, Hallenweg 15, Enschede, 7522 NB, The Netherlands.

*Corresponding author(s). E-mail(s): f.mugele@utwente.nl;
Contributing authors: v.d.siekman@utwente.nl; s.w.reuvekamp@utwente.nl; liuenqing16@gmail.com; s.j.a.debeer@utwente.nl;
†These authors contributed equally to this work.

Keywords: White light interferometry, colorimetry, adaptive surfaces, polymerbrushes, PLMA, dynamic wetting , halo development, controlled evaporation

1 Additional experimental information

1.1 Grafting density estimations

In order to estimate the grafting density of the poly(lauryl methacrylate) (PLMA) brushes and confirm that the films are in the brush regime, knowledge of the polymer molecular weight is required. Direct determination from surface-grafted chains is challenging due to the extremely low total polymer mass. Furthermore, degrafting procedures typically require harsh chemical conditions, which may lead to polymer degradation and consequently unreliable molecular weight determination.

To circumvent these limitations, bulk polymerization was carried out in parallel to the surface-initiated ARGET-ATRP under identical conditions [1]. A small amount of sacrificial initiator, ethyl α-bromoisobutyrate (EBiB), was added to the reaction mixture (0.125 μL, 0.83 μmol), which is chemically analogous to the surface-bound

initiator (BiBB). This very low initiator concentration was chosen since higher concentrations clearly perturbed the surface polymerization, making comparison with brush thickness less reliable. We note that the synthesis still suffered from polymer aggregating and precipitating from the solution, but lower EBiB concentrations would complicate the work-up described below further.

1.1.1 Purification of solution polymer

The polymer was purified to a degree sufficient for gel permeation chromatography (GPC) analysis by first removing all solvents from the original reaction mixture via rotary evaporation. The resulting residue was redissolved in toluene, followed by three successive liquid–liquid extraction steps with deionized water to remove water-soluble species, including copper complexes and residual ascorbic acid.

The remaining toluene phase, containing both polymer and residual monomer, was concentrated by rotary evaporation to yield a viscous solution (approximately 1.5 mL). This solution was subsequently diluted with 1 mL of toluene and rapidly injected into 120 mL of ice-cooled methanol/deionized water (100:20 v/v) under vigorous stirring to induce polymer precipitation. As no immediate solid precipitate formed and only turbidity was observed, the mixture was transferred into 50 mL Falcon tubes and centrifuged for 1.5 h at 7900 rpm. The clear supernatant was decanted, and the resulting white precipitate was redissolved in toluene.

Finally, the solution was concentrated by rotary evaporation, yielding a small amount of slightly viscous, transparent material. To avoid potential material loss during further purification, diffusion-ordered NMR spectroscopy (^{1}H-DOSY NMR) was performed, confirming the presence of both polymer/oligomer and a significant fraction of residual monomer (SI Fig. 2). As the primary objective of this experiment was to estimate the molecular weight, the sample was used directly for GPC analysis, and the presence of residual monomer taken into account in the interpretation of the chromatogram.

1.1.2 GPC analysis

Gel permeation chromatography (GPC) analysis was performed in THF as eluent at 25 °C using a PSS SECcurity system equipped with a refractive index (RI) detector (Waters 515). Separation was achieved using a combination of PSS SDV columns (10000 Å and 50 Å), operated at a flow rate of 0.977 mL min^{-1}. The system was calibrated against polystyrene standards using a third-order polynomial fit. The resulting molar mass distribution (SI Fig. 1) yielded:

$$\begin{aligned} M_{\mathrm{n}} &= 2.29 \times 10^4 \text{ g mol}^{-1} \\ M_{\mathrm{w}} &= 8.17 \times 10^4 \text{ g mol}^{-1} \\ M_{\mathrm{z}} &= 2.95 \times 10^5 \text{ g mol}^{-1} \\ D &= 3.56 \end{aligned}$$

The relatively high dispersity is not unexpected and can be attributed to several factors. First, the presence of residual monomer in the sample contributes to distortions in the GPC trace, particularly affecting the low molecular weight region. Second, the limited solubility of PLMA in ethanol during polymerization plays a dominant role. As the polymer chains grow, they progressively lose solubility, leading to aggregation and precipitation. This results in premature termination of a significant fraction of chains and consequently a broad molecular weight distribution. Additionally, the reaction mixture was not degassed prior to polymerization. Oxygen diffusion from the headspace into the solution can lead to partial deactivation of the catalyst, particularly near the solution–air interface. While surface-initiated polymerization is less affected due to its location within the bulk solution, chains formed in solution are more susceptible to oxygen-induced termination, further broadening the molecular weight distribution. We therefore focus on the data that indicate that high molecular weights are accessible under the employed conditions, as reflected by the large M_z value. This suggests that a fraction of chains can grow to high degrees of polymerization before termination occurs.

1.2 Estimations of grafting density

The grafting density ρ_g can be estimated using:

$$\rho_\mathrm{g} = \frac{H_\mathrm{dry} N_\mathrm{A} \rho}{M} \tag{1.1}$$

where H_dry is the dry brush thickness, N_A Avogadro's number, $\rho = 0.929$ g cm^{-3} is the bulk polymer density, and M is the molar mass of the polymer.

Conventionally, the number-average molecular weight M_n is used. However as elaborated on above, the broad molecular weight distribution render bulk M_n a poor descriptor of the chains contributing to the brush thickness. Instead, we consider M_z more representative of the longer chains that dominate the brush structure.

Using $M_\mathrm{z} = 2.95 \times 10^5$ g mol^{-1} and a measured dry brush thickness of 74 nm, we calculate a grafting density of:

$$\rho_\mathrm{g} \approx 0.14 \text{ chains nm}^{-2}$$

This value lies well within the range typically reported in literature for polymer brushes synthesized via surface-initiated ATRP (SI-ATRP) [1, 2].

As an additional sanity check, we estimate the grafting density at which the polymer chains transition from the mushroom to the brush regime. This transition occurs when the lateral spacing between grafting sites becomes comparable to the size of an isolated polymer coil, and can be approximated as [3]:

$$\rho_\mathrm{g}^* \sim \frac{1}{\pi R_\mathrm{g}^2} \tag{1.2}$$

where R_g is the radius of gyration of the polymer chains, as described in classical polymer brush theory [4].

The radius of gyration can be estimated using standard polymer scaling relations:

$$R_{\rm g} \approx aN^{\nu} \tag{1.3}$$

where a is the Kuhn length, N the degree of polymerization, and ν the Flory exponent. For methacrylate-based polymers, the Kuhn length is typically on the order of $a \approx 1.7$ nm [5]. Considering methacrylate polymers in air, which acts as a poor solvent, $\nu \approx 1/3$ is appropriate [4, 5] .

Using $N \approx 1200$ and $a = 1.7$ nm, this yields:

$$R_{\rm g} \approx 1.7 \times 1200^{0.5} \approx 18 \text{ nm} \tag{1.4}$$

Substituting this into Eq. (2), we obtain:

$$\rho_{\rm g}^{*} \sim \frac{1}{\pi(18)^2} \approx 1 \times 10^{-3} \text{ chains nm}^{-2} \tag{1.5}$$

The experimentally determined grafting density of ~ 0.14 chains nm^{-2} is thus more than two orders of magnitude higher than this threshold. This confirms that the system is well within the polymer brush regime. This conclusion is consistent with classical scaling arguments, where the dimensionless overlap parameter $\Sigma \sim \rho_{\rm g} R_{\rm g}^2 \gg 1$ indicates strong chain stretching [4].

A final estimated grafting density follows from the swelling ratios of brushes with Alexander-de Gennes' theory, which states:

$$\rho_{\rm g} = a \cdot S^{-3/2} \tag{1.6}$$

where a is the statistical monomer length, and S the swelling ratio. We use $a = 0.85$ nm for the statistical monomer length, as described in Rubenstein et al. [5] for methylmethacrylates. Since our polymer brushes swell by a factor of ~ 4 near the droplet contact line and before liquid puddles form on top when infused with vapor, we calculate $\rho_{\rm g} \approx 0.1$ chains nm^{-2}.

Finally, the dimensionless grafting density used in the de Gennes free energy expression of the solvent in the brush is given by $\sigma = \rho_{\rm g} b^2$, where b represents the characteristic size of a lattice site determined from the monomeric volume via the relation $b = (M_0/(\rho_{\rm b} N_{\rm A}))^{1/3} \approx 0.78$ nm with $\rho_{\rm b} = 772$ kg/m^3 the bulk mass density of PLMA and $M_0 = 0.226$ kg/mol the molar mass of the monomer. This value for b is in close agreement with the segment length $a = 0.85$ nm reported by Rubinstein et al. [5]. Using the previously defined $\rho_{\rm g} = 0.1$ nm^{-2}, we estimate the dimensionless density to be $\sigma \approx 0.05$.

1.3 ^{1}H-DOSY NMR

Diffusion Ordered SpectroscopY (DOSY) measurements were performed using a stimulated echo sequence with bipolar sine gradient pulses and eddy current delay before detection (*ledbpgp2s*). Each experiment consisted in acquiring 16 spectra with 16 K data points and a relaxation delay of 3 s. The sine shaped pulsed gradient (g) was incremented from 5% to 95% to the maximum gradient strength in a linear

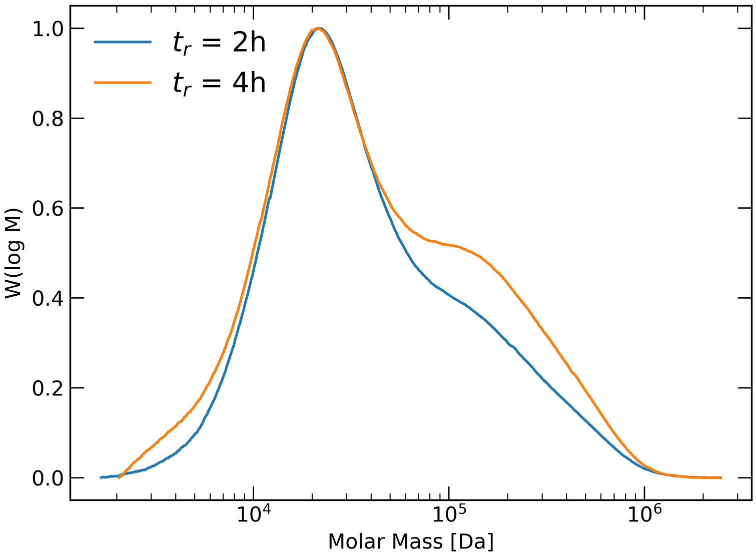

Fig. 1: GPC data of partially purified bulk PLMA polymer grown from sacrificial EBiB initiator, parallel to a BiBB functionalized surface, for (blue) 2 hours and (orange) 4 hours

ramp. The total diffusion-encoding pulse duration (δ) and diffusion delay (Δ) were optimized to 2.64 ms and 60 ms, respectively. The eddy current delay set to 5 ms and the gradient recovery time was 200 μs. The ^{1}H-DOSY data were processed and analyzed using MestRENova 14.1.2-25024 software.

In SI Fig. 2, we present a DOSY measurement of a partially purified PLMA bulk sample (2 h reaction time) in $CDCl_3$. The spectrum confirms the presence of residual monomer (dashed vertical line) alongside a broad distribution of polymer and oligomer species (dashed box). As expected, the monomer exhibits a higher diffusion coefficient due to its smaller size, while the polymer and oligomers show reduced diffusion coefficients that decrease with increasing chain length.

Although the monomer and polymer share similar chemical structures and therefore exhibit overlapping resonances in the chemical shift dimension, they can be clearly distinguished spectroscopically. In particular, the disappearance of the vinyl $C{=}CH_2$ signals at 5.55 and 6.12 ppm, together with the shift and broadening of the $-OCH_2-$ triplet from 4.17 ppm to ~3.93 ppm, confirms successful polymerization. The spread in diffusion coefficients further indicates a broad molecular weight distribution.

1.4 XPS wide spectrum

The wide-scan XPS of the poly(lauryl methacrylate) polymer brush on silicon in SI Fig. 3 shows dominant C 1s (90.8 at%), O 1s (8.5 at%) and minor Si 2s&2p (0.7 at%)

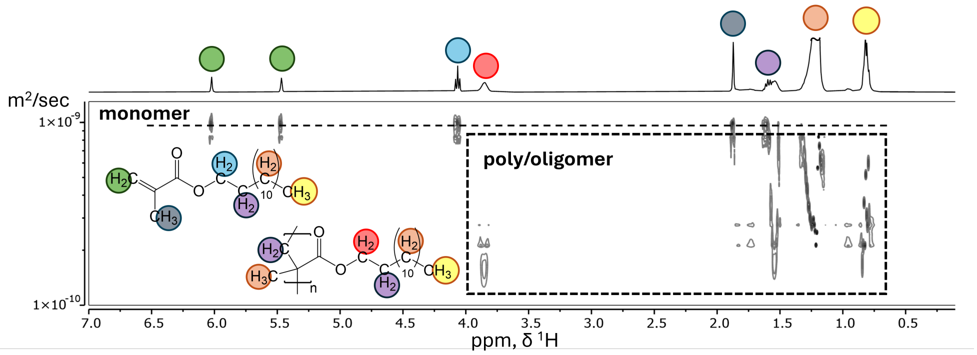

Fig. 2: ^{1}H-DOSY NMR of partially purified bulk PLMA polymer grown from sacrificial EBiB initator (2 hours) in chloroform-d. The dashed line represents the monomer, the dashed box the polymer and oligomer.

signals. The nominal atomic composition of the PLMA repeat unit $C_{16}O_2$ (O:C = 1:8). The observed O:C ratio of 1:11 is slightly lower than the expected stoichiometry, which we attribute to absorption of adventitious atmospheric carbon [6] and polymer degradation of the oxygen-functional groups. We observed the oxygen content decrease under prolonged exposure to the X-ray beam and the applied dual-beam charge-neutralization electrons, as also reported in [7]. Despite these effects, the overall composition is consistent with the presence of a poly(lauryl methacrylate) polymer brush on silicon.

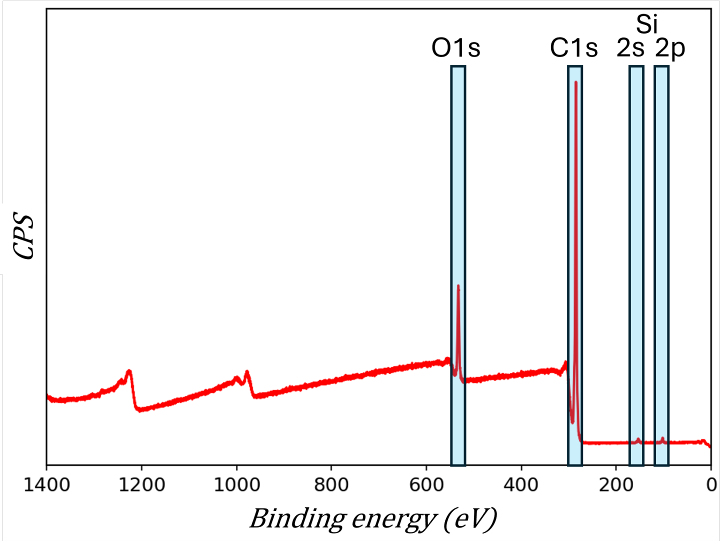

Fig. 3: Wide XPS spectrum of poly-lauryl methacrylate

2 n-alkane solvent properties

Tables 1 and 2 list the DIPPR 5-parameter coefficients for the saturation pressures P^n_{sat} and dynamic viscosities μ^n of n-dodecane, n-tetradecane, and n-hexadecane [8]. Both properties can be calculated using the empirical form:

$$\ln y = C_1 + \frac{C_2}{T} + C_3 \ln T + C_4 T^{C_5}$$

where y represents P^n_{sat} or μ^n. Based on these correlations, Table 3 summarizes the key transport properties at $T = 298.15$ K including P_{sat}, μ, the self-diffusion coefficient D_{self}, and their respective ratios to facilitate a comparison of key solvent transport properties across the homologous series.

**Solvent**	C_1	C_2	C_3	C_4	C_5	T_{min}	P **at** T_{min}	T_{max}	P **at** T_{max}
n-$C_{12}H_{26}$	137.47	-11976	-16.698	8.0906E-06	2	263.57	6.15E-01	658	1.822E+06
n-$C_{14}H_{30}$	140.47	-13231	-16.859	6.5877E-06	2	279.01	2.53E-01	693	1.569E+06
n-$C_{16}H_{34}$	156.06	-15015	-18.941	6.8172E-06	2	291.31	9.23E-02	723	1.411E+06

Table 1: DIPPR Vapor Pressure Parameters and range limits for n-alkanes from [8].

**Solvent**	C_1	C_2	C_3	C_4	C_5	T_{min}	μ **at** T_{min}	T_{max}	μ **at** T_{max}
$C_{12}H_{26}$	-7.8244	1191.9	-0.49963	3.9572×10^{23}	-10	262.15	3.020E-03	526.40	1.680E-04
$C_{14}H_{30}$	-14.493	1710.8	0.4417	3.0895×10^{28}	-12	277.65	3.350E-03	554.40	1.810E-04
$C_{16}H_{34}$	-20.182	2203.5	1.2289	5.6884×10^{21}	-10	291.31	3.536E-03	564.15	2.054E-04

Table 2: DIPPR Liquid viscosity parameters and range limits for n-alkanes from [8].

**Solvent**	P_{sat} [Pa]	P^{n-2}_{sat}/P_n	μ [mPa·s]	μ_{n-2}/μ_n	$D^{\text{L}}_{\text{self}}$ [10^{-9} m^2/s]	D_n/D_{n-2}	$\mu \cdot D$ [10^{-12} N]	D_{v} [10^{-6} m^2/s]
n-$C_{12}H_{26}$	17.868	—	1.349	—	0.814	—	1.155	5.1
n-$C_{14}H_{30}$	1.864	9.586	2.068	0.652	0.520	0.639	1.160	4.5
n-$C_{16}H_{34}$	0.199	9.367	3.062	0.675	0.376	0.723	1.151	4.0

Table 3: Saturation pressures and transport properties for n-alkanes of interest, evaluated at $T = 298.15K$ [8–10].

3 Deswelling of pre-swollen PLMA brushes

3.1 Details on evaporation data-analysis

Tables 4, 5, and 6 summarize the analyzed data from the deswelling experiments discussed in the main text (Fig. 6) for dodecane ($C12$), tetradecane ($C14$), and hexadecane ($C16$), respectively. The tables list the evaporation rates $\bar{k}$ (reported in both 1/s and 1/min) extracted from the linear portion of the deswelling curves. Each value follows from an averaging of at least three independently measured experiments per initial swelling ratio.

Along with these rates, for each solvent we report the initial average swollen thickness $\bar{h}$ and the corresponding initial swelling ratio $\bar{\alpha}_0 = \bar{h}/h_0$, where the dry thickness h_0 is held constant between 165–175 nm. Additionally, the tables include the time shift t_0 and the reported uncertainties σ, which reflect the standard deviation across all available data points in each category. From these averaged rates, we calculate the following ratios:

$$\frac{\bar{k}^{C14}}{\bar{k}^{C16}} = \frac{24.41}{2.13} \approx 11.46, \quad \frac{\bar{k}^{C12}}{\bar{k}^{C14}} = \frac{193.34}{24.41} \approx 7.92 \tag{3.1}$$

These ratios closely align with the corresponding saturation pressure ratios provided in Table 3. For instance:

$$\frac{\bar{k}^{C12}}{\bar{k}^{C16}} = \frac{193.34}{2.13} = 90.77 \quad \frac{P_{\text{sat}}^{C12}}{P_{\text{sat}}^{C16}} = 89.79 \tag{3.2}$$

This strong agreement indicates that the measured evaporation rates accurately reflect the relative volatilities of the three n-alkanes.

Table 4: Evaporation data $C_{12}H_{26}$

Index	$\bar{h}$ (nm)	$\bar{\alpha}_0$	k $(1/s)$	k $(1/min)$	t_0 (s)
1, blue	291	1.66	3.29	197.41	166,00
2, black	433	2.47	2.88	173.05	117.00
3, red	550	3.14	3.41	204.85	82.00
4, green	627	3.58	3.14	188.41	45.00
5, cyan	755	4.32	3.38	202.97	0.00
$\sum y_i/N$			3.22 ± 0.22	193.34 ± 13.02	

3.2 Deswelling kinetics for dodecane and hexadecane

Following the same procedure described in the main text, we compare the calculated deswelling trends for dodecane and hexadecane with the experimental datasets (SI Fig. 4). The experimental data (gray circles) correspond to the bin-averaged values derived from the full set of results presented in Fig. 6 of the main text, with error bars

Table 5: Evaporation data $C_{14}H_{30}$

Index	$\bar{h}$ (nm)	$\bar{\alpha}_0$	k $(1/s)$	k $(1/min)$	t_0 (s)
1, blue	232.83	1.33	4.30×10^{-1}	25.77	1150.00
2, black	414.06	2.37	3.17×10^{-1}	19.02	650.00
3, red	492.81	2.82	3.86×10^{-1}	23.16	509.58
4, green	663.73	3.79	4.61×10^{-1}	27.68	213.30
5, cyan	748.48	4.28	4.40×10^{-1}	26.40	0.00
$\sum x_i/N$			$(4.07 \pm 0.57) \times 10^{-1}$	24.41 ± 3.43	

Table 6: Evaporation data $C_{16}H_{34}$

Index	$\bar{h}$ (nm)	$\bar{\alpha}_0$	k $(1/s)$	k $(1/min)$	t_0 (s)
1, blue	263.64	1.51	2.40×10^{-2}	1,44	8147.20
2, black	375.99	2.15	3.63×10^{-2}	2,17	5749.90
3, red	433.30	2.48	3.61×10^{-2}	2.17	4268.00
4, green	543.84	3.11	4.33×10^{-2}	2,60	2153.80
5, cyan	600.62	3.43	3.81×10^{-2}	2.29	0,00
$\sum x_i/N$			$(3.56 \pm 0.71) \times 10^{-2}$	2.13 ± 0.43	

representing the standard deviation across all measurements. As shown in SI Fig. 4, the theoretical relation:

$$\dot{\alpha} = -\frac{DP_{\text{sat}}}{kTL\rho_S h_0} a(\alpha) = -K \exp(f(\alpha)) \tag{3.3}$$

integrated with the same initial conditions ($\alpha(0) = 4.25$), provides an excellent fit for both solvents when treating the kinetic prefactor K as an adjustable parameter. These best-fit values, summarized in Table 7, scale directly with the alkane volatility; notably, the ratio K/P_{sat} remains approximately constant, varying by less than a factor of two despite a nearly hundred-fold difference in saturation pressure.

From the kinetic prefactor K, we can calculate the characteristic thickness of the stagnant gas layer L by:

$$L = \frac{D_v P_{\text{sat}}}{k_B T \rho_S h_0 K} \tag{3.4}$$

where the solvent number densities, $\rho_S = \rho_m N_A / M_w$, are 2.65×10^{27}, 2.33×10^{27}, and 2.08×10^{27} molecules/m^3 for dodecane, tetradecane, and hexadecane, respectively, are calculated via the solvent mass density ρ_m. As summarized in Table 7, the resulting values for L fall within the millimeter range $(2 - 4$ mm), consistent with the expected macroscopic distance over which solvent vapor must diffuse into the ambient environment.

Using the mean stagnant layer thickness, $L \approx 2.70$ mm, we compare the characteristic timescales for vapor-phase diffusion and liquid-phase diffusion within the brush.

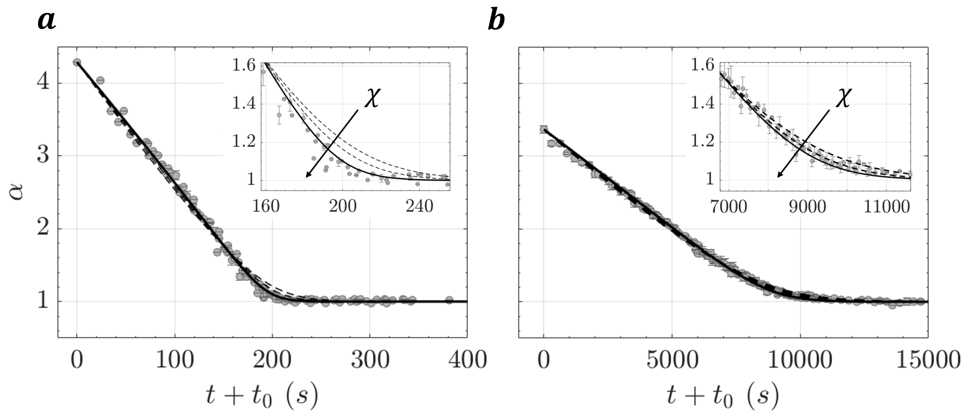

Fig. 4: Bin-averaged experimental data (gray circles) and theoretical fits (solid lines) for **a)** dodecane ($C12$) and **b)** hexadecane ($C16$). Error bars represent the standard deviation across all measurements. The theoretical curves are obtained by integrating the kinetic relation described in the main text, using K as the fitting parameter. The insets provide a magnified view of the regime where $a(\alpha) < 1$ ($1 < \alpha < 1.6$); here, the calculated curves shift from the outer dashed line ($\chi = 0$) toward the solid line ($\chi = 0.7$), with $\chi = 0.4$ shown as the intermediate dashed line.

Taking tetradecane as a representative case ($D_\mathrm{v} \approx 4.5 \times 10^{-6}$ m^2/s) and approximating the solvent diffusion coefficient within the polymer brush as $D_\mathrm{br} \approx 10^{-10}$ m^2/s [11] with $h_0 = 175$ nm, the respective timescales are:

$$\tau_\mathrm{v} \approx \frac{L^2}{D_\mathrm{v}} \approx 1.62 \text{ s}, \quad \tau_\ell \approx \frac{h_0^2}{D_\mathrm{br}} \approx 3.1 \times 10^{-4} \text{ s} \tag{3.5}$$

The separation of more than three orders of magnitude between these timescales ($\tau_\mathrm{v}/\tau_\ell \approx 5.3 \times 10^3$) confirms that deswelling is strictly rate-limited by the transport of solvent vapor through the stagnant gas layer, ensuring a spatially uniform solvent concentration within the brush and justifying a quasi-equilibrium modeling approach.

Table 7: Kinetic parameters and effective diffusion path lengths L for the studied n-alkanes.

**Solvent**	P_sat [Pa]	K [s^{-1}]	K/P_sat [s^{-1} Pa^{-1}]	L [mm]
n-$C_{12}H_{26}$	17.87	1.31×10^{-2}	7.32×10^{-4}	3.64
n-$C_{14}H_{30}$	1.86	2.13×10^{-3}	1.14×10^{-3}	2.35
n-$C_{16}H_{34}$	0.20	2.55×10^{-4}	1.28×10^{-3}	2.09
Mean			$(1.05 \pm 0.28) \times 10^{-3}$	2.70 ± 0.83

3.3 Influence of grafting density on deswelling behavior

To assess how the theoretical fits depend on the chosen grafting density, we examine the deswelling profiles over a broad range of σ values. At a constant $\chi = 0.7$, higher grafting densities (e.g., $\sigma = 0.5$) introduce a pronounced curvature (blue curves, SI Fig. 5a) that bends away from the linear regime observed experimentally ($1.6 \lesssim \alpha \lesssim 4.2$). While the dashed lines in SI Fig. 5a illustrate that increasing χ from 0 to 1 shifts the theoretical curves toward the data, the experimental trend can only be matched by unrealistically large Flory–Huggins parameters ($\chi \gg 1$).

The discrepancy is further quantified in SI Fig. 5b for a constant interaction parameter $\chi = 0.7$, where the root mean square error ($|\Delta E|$) increases sharply for $\sigma > 0.15$. Consequently, our estimate of the grafting density based on the characteristic size of a lattice site, $\sigma = \rho_{\mathrm{g}} b^2 \approx 0.05$ (see preceding section), appears to provide a physically realistic description of the elastic properties of the swollen brush.

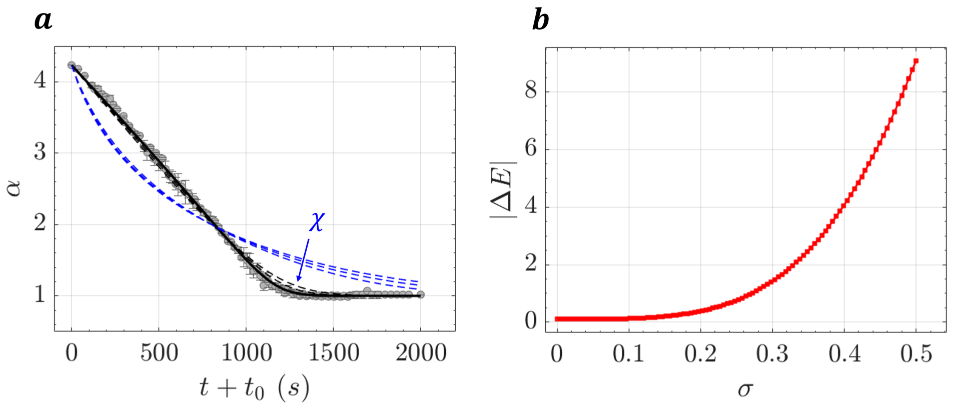

Fig. 5: **a**) Bin-averaged deswelling data (C_{14}, gray circles) plotted together with calculated curves (black lines) for $\sigma = 0.05$ and $\chi = 0$–0.7, as described in the main text. The blue curves illustrate the strong curvature in the deswelling response for $\sigma = 0.5$, deviating from the experimentally observed linear behavior; dashed lines correspond to $\chi = 0$–1. **b**) Root mean square error, $|\Delta E|$, as a function of grafting density, showing a strong increase in deviation from the experimental dataset for $\sigma > 0.15$.

4 Halo solvent content

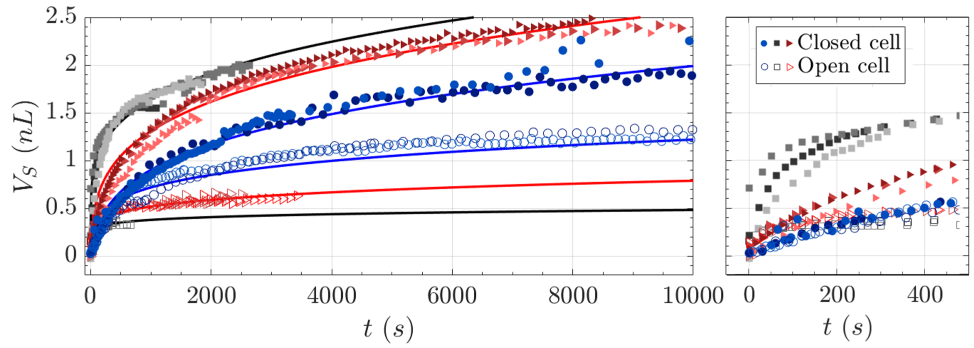

Fig. 6: Solvent volume $V_s(t)$ in the halo as a function of time for dodecane (black, □), tetradecane (red, △) and hexadecane (blue, ○). Open symbols show the oil content in the open-cell configuration, while filled symbols represent the closed-cell configuration. Note the mirrored pattern, where the difference in oil content between the open and closed configuration decreases with increasing alkane chain length. The inset shows a zoomed in version of the initial behavior, which demonstrates the fast initial swelling of smaller n-alkanes.

From the brush profiles (Fig. 8, main text), we calculate the solvent volume in the halo, $V_{\mathrm{S}}(t)$ as a function of time (SI Fig. 6) for dodecane (black, □), tetradecane (red, △) and hexadecane (blue, ○). Open symbols show the oil content in the open-cell configuration, while filled symbols represent the growth in the closed-cell configuration. To provide a sense of scale, for a typical droplet radius $r_{\mathrm{drop}} \approx 2.0$ mm after initial spreading (where $\theta = \theta_{\mathrm{eq}}$), the halo region extending 1.0 mm beyond the contact line can accommodate a maximum solvent volume of $V_{\mathrm{S}}^{\mathrm{max}} \approx 9.0$ nL, estimated from the area of this annulus multiplied by the maximum swelling height difference $\Delta h = h_{\mathrm{eq}} - h_0$ between a dry and fully swollen brush. Consistent with the observed profiles, the solvent volume in the open cell approaches a plateau, with the final halo volume for hexadecane $V_{\mathrm{S}}^{C16} = 1.37$ nL being much larger than that for dodecane $V_{\mathrm{S}}^{C12} = 0.32$ nL (see inset for early-time behavior). In contrast, the solvent volume in a closed cell configuration increases more rapidly, leading to an mirrored pattern in which the halo volume for dodecane now exceeds that for hexadecane. The algebraic fits serve as guides to the eye, illustrating that halo development in the closed cell has not yet saturated and would, in principle, continue until the brush reaches its fully swollen state or till the droplet is exhausted. From the data in the figure, we find that in the limit of large n-alkanes, the difference in solvent accumulation between open- and closed cell minimizes due to the negligible of vapor transport, whereas for short-chain alkanes, rapid evaporation in the open cell strongly limits swelling, while the reduced evaporation and enhanced condensation in the closed cell allow much more extensive halo development.

References

[1] Matyjaszewski, K., Dong, H., Jakubowski, W., Pietrasik, J., Kusumo, A.: Grafting from surfaces for "everyone": Arget atrp in the presence of air. Langmuir **23**(8), 4528–4531 (2007) https://doi.org/10.1021/la063402e

[2] Zoppe, J.O., Ataman, N.C., Mocny, P., Wang, J., Moraes, J., Klok, H.-A.: Surface-initiated controlled radical polymerization: State-of-the-art, opportunities, and challenges in surface and interface engineering with polymer brushes. Chemical Reviews **117**(3), 1105–1318 (2017) https://doi.org/10.1021/acs.chemrev.6b00314

[3] Biesalski, M., Rühe, J.: Scaling laws for the swelling of neutral and charged polymer brushes in good solvents. Macromolecules **35**(2), 499–507 (2001) https://doi.org/10.1021/ma001776n

[4] De Gennes, P.-G.: Scaling Concepts in Polymer Physics. Cornell university press, New York (1979)

[5] Rubinstein, M., Colby, R.H.: Polymer Physics. Oxford university press, New York (2003)

[6] Giglio, E., Ditaranto, N., Sabbatini, L.: 3. Polymer surface chemistry: Characterization by XPS, pp. 73–112. DE GRUYTER, Berlin (2014)

[7] Svoboda, J., Sivkova, R., Dorado Daza, D.F., de los Santos Pereira, A., Pop-Georgievski, O.: Xps analysis of zwitterion polymer brushes: discrepancies, degradation and methodological considerations. Applied Surface Science **722**, 165479 (2026) https://doi.org/10.1016/j.apsusc.2025.165479

[8] Perry, J.H.: Chemical engineers' handbook. ACS Publications (1950)

[9] Marrero, T.R., Mason, E.A.: Gaseous diffusion coefficients. Journal of Physical and Chemical Reference Data **1**(1), 3–118 (1972) https://doi.org/10.1063/1.3253094

[10] Holz, M., Heil, S.R., Sacco, A.: Temperature-dependent self-diffusion coefficients of water and six selected molecular liquids for calibration in accurate 1h nmr pfg measurements. Physical Chemistry Chemical Physics **2**(20), 4740–4742 (2000) https://doi.org/10.1039/b005319h

[11] Kap, Ö., Hartmann, S., Hoek, H., de Beer, S., Siretanu, I., Thiele, U., Mugele, F.: Nonequilibrium configurations of swelling polymer brush layers induced by spreading drops of weakly volatile oil. The Journal of chemical physics **158**(17) (2023) https://doi.org/10.1063/5.0146779
